# A Self‐Powered Piezo‐Bioelectric Device Regulates Tendon Repair‐Associated Signaling Pathways through Modulation of Mechanosensitive Ion Channels

**DOI:** 10.1002/adma.202008788

**Published:** 2021-08-23

**Authors:** Marc A. Fernandez‐Yague, Alexandre Trotier, Secil Demir, Sunny Akogwu Abbah, Aitor Larrañaga, Arun Thirumaran, Aimee Stapleton, Syed A. M. Tofail, Matteo Palma, Michelle Kilcoyne, Abhay Pandit, Manus J. Biggs

**Affiliations:** ^1^ CÚRAM SFI Research Centre for Medical Devices National University of Ireland Galway H91W2TY Ireland; ^2^ University of the Basque Country Department of Mining‐Metallurgy Engineering and Materials Science and POLYMAT Barrio Sarriena Bilbao 48013 Spain; ^3^ University of Limerick Department of Physics Limerick V94 T9PX Ireland; ^4^ Queen Mary University of London Materials Research Institute and School of Biological and Chemical Sciences Mile End Road London E1 4NS UK

**Keywords:** bioelectronics, collagen, piezoelectrics, poly(vinylidene fluoride‐*co*‐trifluoroethylene), tendon regeneration

## Abstract

Tendon disease constitutes an unmet clinical need and remains a critical challenge in the field of orthopaedic surgery. Innovative solutions are required to overcome the limitations of current tendon grafting approaches, and bioelectronic therapies show promise in treating musculoskeletal diseases, accelerating functional recovery through the activation of tissue regeneration‐specific signaling pathways. Self‐powered bioelectronic devices, particularly piezoelectric materials, represent a paradigm shift in biomedicine, negating the need for battery or external powering and complementing existing mechanotherapy to accelerate the repair processes. Here, the dynamic response of tendon cells to a piezoelectric collagen‐analogue scaffold comprised of aligned nanoscale fibers made of the ferroelectric material poly(vinylidene fluoride‐*co*‐trifluoroethylene) is shown. It is demonstrated that motion‐powered electromechanical stimulation of tendon tissue through piezo‐bioelectric device results in ion channel modulation in vitro and regulates specific tissue regeneration signaling pathways. Finally, the potential of the piezo‐bioelectronic device in modulating the progression of tendinopathy‐associated processes in vivo, using a rat Achilles acute injury model is shown. This study indicates that electromechanical stimulation regulates mechanosensitive ion channel sensitivity and promotes tendon‐specific over non‐tenogenic tissue repair processes.

## Graphical abstract

Under physiological conditions, ion‐channels activity limit membrane depolarization and regulate ion concentration‐dependent signaling cascades. In response to injury or mechanical loading, the activity and expression of the ion‐channels increases, and unspecific tissue‐repair signaling pathways activate, resulting in ectopic calcification. By modulating the mechanosensitive ion‐channels’ expression, electromechanical stimulation regulates specific signaling pathways activation to promote tendon over bone formation processes.

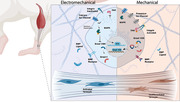

## 1 Introduction

Severe tendon injuries resulting from athletic or repetitive activity affect more than 102.5 million adults every year and place a considerable burden on healthcare systems (> $2 billion annually, and post‐surgery complications result in nearly 1 million additional days of inpatient care each year).^[^ ^1^, ^2^, ^3^ ^]^ Surgical intervention via direct end‐to‐end repair using sutures and biological or synthetic grafts represents the gold standard in treatment, and despite the relative success, these repairs frequently fail to restore full tendon functionality. Following injury, disorganized tissue deposition leads to scar tissue formation, proteoglycan accumulation, and calcification, resulting in poor biomechanical properties and impaired function that triggers chronic inflammatory signaling pathways and progresses into tendinopathy. Hence, to achieve long‐term repair, innovative functional solutions that focus on the activation of endogenous tissue‐repair signaling pathways represents a paradigm shift in the field of biomedical devices and regenerative medicine (RM).^[^ ^4^ ^]^

**Figure 1:**
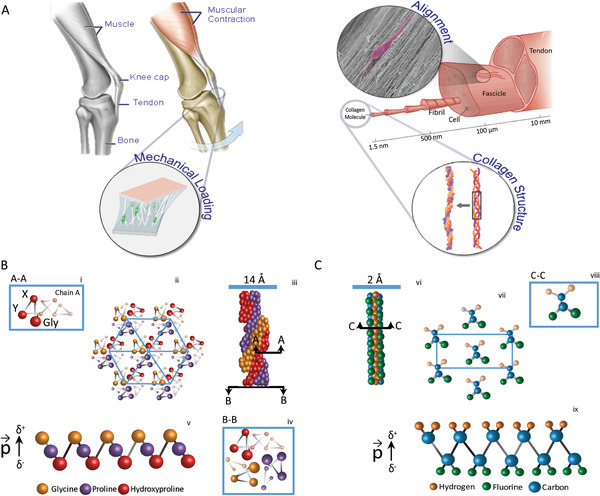
Overview of the tendon electromechanical environment and the piezoelectricity origin analogy of collagen and PVDF‐TrFE fibers. Tendon is a dynamic tissue that connects muscle to bone and is under continuous mechanical loading and unloading. A) This mechanical stress is borne by a highly anisotropic extracellular matrix composed of collagen type I, a high‐tensile, piezoelectric material which undergoes electrical polarization in response to mechanical loading. B) Schematic representation of the crystal structure of the collagen triple helix consisting of three polypeptides chains stabilized via hydrogen bonding. A‐A: Cross‐sectional schematic of an individual alpha‐chain showing the triple Gly (glycine)‐X (proline)‐Y (hydroxyproline) residuesi (i). Polar bonds between carbonyl (CO groups) and amine (peptide NH group of glycine residues) groups along the backbone of collagen result in a dipole moment or electrical polarization (ii). Cross‐section of multiple individual collagen molecules resulting in a non‐centrosymmetric hexagonal fibril arrangement (iii). B‐B: A cross‐section of the collagen triple‐helix core; here, the three helical chains and glycine residues are observable (iv). Representation of an individual electric dipole (v). C) Representation of the PVDF‐TrFE all‐trans (TTTT) zigzag planar configuration top view (vi). Cross‐section of the crystal and along the orthogonal axis of the all‐trans (TTTT) zigzag planar configuration (vii). Representation of an individual electric dipole composed of fluorine (green sphere), carbon (blue sphere), and hydrogen (orange sphere) atoms (viii). PVDF‐TrFE has a non‐centrosymmetric structure. Dipoles are generated by the highly electronegative difference between hydrogen and fluorine atoms. Representation of the molecular chain structure of the electroactive β‐phase of PVDF‐TrFE showing a resultant dipole moment (ix).

Many studies confirm that resident tendon cell populations are highly mechanosensitive and are responsible for orchestrating the repair processes after injury through specialized sensory machinery, including mechanosensitive ion channels.^[^ ^5^, ^6^, ^7^, ^8^ ^]^ Critically, mechanotherapy (i.e., low‐level exercise or extracorporeal shock waves) has been reported to promote tendon regeneration and provide a reliable route for appropriate postoperative management.^[^ ^9^ ^]^ Additionally, piezoelectricity‐derived electric fields produced during physiological locomotion may provide additional bioelectric signaling cues to activate tendon‐specific regenerative pathways (**Figure** **1**A). Several recent studies have revealed the significant potential of electrical fields in mediating cell migration, promoting collagen synthesis, and inducing successful wound healing by externally applied direct current electrical stimulation (ES).^[^ ^10^, ^11^ ^]^ In the last decade, DC stimulators (i.e., Zimmer Biomet SpF and OsteoGen) have improved the success rate of spinal fusion procedures and non‐healing tissue injuries.^[^ ^12^ ^]^ Despite the proven clinical effect, a significant roadblock to wide‐scale clinical adoption of ES is the infection risk to penetrating electrodes, potential non‐specific off‐target effects, and the cumbersome design of ES units, making patient compliance a concern.^[^ ^13^ ^]^ The need for more safe, efficient, and less invasive electrically stimulating systems drives the development of novel bioelectric strategies that control somatic cell functions and enhance specific tissue regenerative processes.^[^ ^14^ ^]^

The discovery of piezoelectricity in bone and more recently in its constituent collagen type I, has spurred new research into the role of bioelectricity in tissue regeneration and the development of self‐powered electrical stimulation technologies.^[^ ^15^, ^16^ ^]^ A promising strategy for developing biomimetic electromechanical stimulation (EMS) devices has been enabled by synthesizing compliant ferroelectric polymers (i.e., PVDF‐TrFE), conformed into a nanofibrous scaffold that mimics the intrinsic electrical, mechanical, and morphological properties of collagen type I (Figure 1B). Mechanical actuation of these structures or piezo‐bioelectric devices through repetitive physiological loading and unloading (i.e., monotonic stretching) can produce biologically relevant electrical fields, enabling studies into the role of “mechanically‐induced” electrical cues on musculoskeletal tissue function. Significant efforts from the biomedical community have demonstrated the potential of piezoelectric materials to transduce mechanical into physiologically relevant electrical cues (direct piezoelectricity) and affect different cell types, including osteoblasts, mesenchymal stem cells, and neurons.^[^ ^17^, ^18^, ^19^, ^20^ ^]^ In this study, we hypothesized that a ferroelectric scaffold could serve as mechanical support for the regeneration of damaged tendon tissues and mimic the bioelectrical cues usually provided by collagen's piezoelectricity to maintain tendon‐cell phenotype and promote tendon regeneration (Figure 1B,C).

## 2 Design and Fabrication of a Piezo‐Bioelectric Device

**Figure 2:**
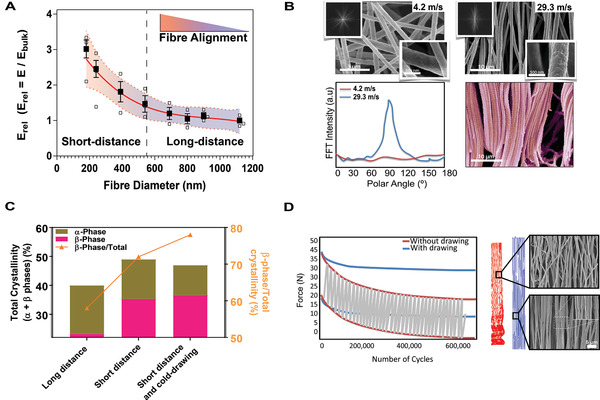
Cold drawing improves fiber alignment and enhances electroactive β‐phase formation. A) Analysis of the relationship between fiber diameter and the relative Young's modulus (Erel) indicated that the mechanical behavior of highly aligned scaffolds was inversely proportional to fiber diameter. B) The morphology of scaffolds obtained by electrospinning collected at low (4.2 m s^−1^ rpm) and high (29.3 m s^−1^) linear speeds. Fast fourier transform (FFT) spectra showed a broad distribution of intensities for low speed (4.2 m s^−1^) and a clear peak for high speed (29.3 m s^−1^) characteristic of highly anisotropic structures. Fiber alignment was significantly increased, and the morphology mimicked that of tendon collagen. C) Total crystallinity and β‐phase content (calculated from FTIR and XRD spectra) increased as a function of collector distance and following cold drawing. D) Creep/stress relaxation was minimized after cold drawing and resulted in reduced fiber diameter and increased fiber alignment. SEM images of scaffolds with and without cold drawing.

The physical properties of a scaffold, including its mechanical behavior, microstructure (fiber diameter and alignment), and crystallinity‐dependent piezoelectric response, play a pivotal role in regulating the cellular events involved in tissue regeneration.^[^ ^21^ ^]^ To develop a collagen piezoelectrical‐analog device that can recapitulate the fibrous structure of extracellular tendon tissue an electrospinning process was implemented, and nanoscale fibers with varying diameter and alignment were produced through optimization of solution and processing parameters (**Figure** **2**).

First, we analyzed the impact of fiber diameter on the biophysical properties of the scaffolds. By varying the concentration of the polymeric solution (1.1, 1.2, 1.3, 1.4, 1.5, 1.6, and 1.7 mg mL^−1^ in dimethylacetamide (DMAc) we could obtain fibers with distinct diameters. We used a 30 kV potential difference between collector and nozzle tip (Ø = 200 µm), a flow rate of 1 mL h^−1^, and a tip‐to‐collector distance of 6 cm (short‐distance electrospinning). Importantly, a collector disk with an 8 mm width was used to concentrate the electrical field while rotating at 29.3 m s^−1^ (linear speed) to draw and align the collected fibers. The resulting fibers ranged from 180 to 540 nm in diameter (Figure 2A; Figure S1, Supporting Information), which is similar to the diameter of collagen fibers in tendon tissue 50–500 nm.^[^ ^22^ ^]^

It was observed that the fiber diameter governed the stiffness of the resulting scaffolds and constructs with lower fiber diameters possessed an increased Young's modulus, as determined by tensile‐test analysis (Figure 2A). The observed enhancement of the mechanical properties of scaffolds with decreasing fiber diameter was a result of a high level of chain extension and orientation along the fiber axis as a consequence of fibers being drawn by centripetal forces during fiber collection. With semi‐crystalline polymers (including poly(vinylidene fluoride‐*co*‐trifluoroethylene) P(VDF‐TrFE)), it has been demonstrated that stress hardening does not depend on polymer crystallinity but rather, on the density of amorphous polymer chain entanglement, which in respect to electrospinning, is related to the distance between the needle and collector, the collector rotational speed and the polymer solution concentration.^[^ ^23^ ^]^ As a result, scaffolds with a fiber diameter between 390 and 540 nm (Figure S1>D,E, Supporting Information) demonstrated increased fiber alignment. Finally, nanofibers with diameters <240 nm (Figure S1F, Supporting Information) were prone to alignment disorganization due to the air currents generated during collection.

To decouple the effect of piezoelectric stimulation from mechanical loading on the cellular response, a non‐piezoelectric PTFE scaffold which maintained the topography (i.e., fiber diameter and organization) and was chemically analogous (i.e., a fluorinated polymer) to PVDF‐TrFE was also fabricated. PTFE is non‐piezoelectric due to the presence of a centrosymmetric structure with strong polar C—F covalent bonds (C*∂*+—F*∂*−). Conversely, PVDF‐TrFE presents non‐centrosymmetric electrical dipoles, generated by the high electronegative difference between hydrogen and fluorine atoms (Figure S2, Supporting Information). The direct electrospinning of PTFE fibers, however, was not possible due to the unique chemical properties of PTFE. Therefore, a two‐step short‐distance electrospinning process was optimized to obtain scaffolds of PTFE fibers.^[^ ^24^ ^]^

The tendon microstructure is highly hierarchical, with collagen fibers aligning to the direction of mechanical loading. To reproduce this critical feature in both PVDF‐TrFE and control PTFE scaffolds the collector velocity was adjusted between 4.2–29.3 m s^−1^, and the concentration of the polymeric solution fixed at 1.7 mg mL^−1^. As evident in Figure 2B, the scaffolds obtained at a collector linear velocity of 29.3 m s^−1^ (≈4000 rpm) displayed a highly organized fiber morphology, while a broad distribution of fiber orientation was observed for scaffolds formulated with a collector linear velocity of 4.2 m s^−1^ (500 rpm). Additionally, as determined by X‐ray diffractometry (Figure S3, Supporting Information and Table S1), the degree of crystallinity increased from 40% in randomly aligned PVDF‐TrFE fibers to 49% in aligned fibers (Figure 2C), suggesting that mechanical drawing during fiber collection at high rotational speeds resulted in PVDF‐TrFE fibers with a reduced diameter (590 ± 130 nm for 4.2 m s^−1^ and 540 ± 120 nm for 29.3 m s^−1^) and enhanced crystallinity (Table S1, Supporting Information). Our short‐distance electrospinning configuration contrasted to previously reported electrospinning configurations for PVDF‐TrFE (i.e., long‐distance electrospinning).^[^ ^25^ ^]^ We have demonstrated that scaffolds produced using a short‐distance configuration possessed increased fiber organization (Figure S1, Supporting Information) and crystallinity (Figure 2C), supporting the hypothesis that fibers are significantly drawn when subjected to an electric‐field increased intensity. The mechanical behavior of the scaffolds obtained by the short‐distance configuration was improved compared to scaffolds obtained by a conventional (long‐distance) configuration in terms of elastic modulus, creep resistance, and yield strength (Figure S2, Supporting Information), which is of vital importance for the device to retain the integrity of its macro and microstructure under loading following implantation.^[^ ^26^ ^]^

Regarding the piezoelectric response, a growing body of research suggests that the piezoelectric performance of PVDF (i.e., the base comonomer of the PVDF‐TrFE copolymer) is affected by the degree of crystallinity and in particular the β‐phase content (Table S2, Supporting Information).^[^ ^27^ ^]^ As determined by Fourier‐transform infrared spectroscopy (FTIR) (Table S2, Supporting Information), the short‐distance configuration promoted the crystallization of the electroactive β‐phase (Figure 2C). Overall, these observations reinforce the argument for the potential use of short‐distance electrospinning in the fabrication of PVDF‐TrFE‐based scaffolds.

To further increase the mechanical properties and β‐phase content of the scaffolds, while avoiding the well‐reported crimping of the fibers under repetitive mechanical loading, two post‐synthesis processes were investigated: i) strain‐hardening (cold‐drawing) and ii) thermal annealing (cold crystallization). Based on the initial properties of the scaffolds, we explored cold drawing at ≈12% to induce strain‐hardening through plastic deformation of the structures. Overall, this process resulted in ≈8% plastic deformation, alignment of fibers (from 65% to 98%) and reduction in individual fiber diameter (from 540 to 516 nm). Importantly, fiber diameter reduction and better fiber alignment resulted in scaffolds with an improved elastic modulus (61.8 ± 5.1 MPa, *p* < 0.05) and strength (31 ± 4.2 Mpa, *p* < 0.001) (**Table** **1**). We conducted extensive SEM inspections along the entire obtained scaffolds (0.8 × 30 cm) to check the fiber direction. We observed that fibers were aligned with the collector rotating direction and parallel to the longitudinal axis of the scaffold. Only a few fibers far from the middle part of the scaffold presented a skewed orientation (<7 ± 6°) and were corrected before implantation of the devices during the subsequent cold drawing process.

**Table 1:** The physical properties of piezoelectric PVDF‐TrFE and non‐piezoelectric PTFE scaffolds

Scaffold type	Elastic modulus [MPa]	Strength [MPa]	Elongation [%]	Fiber diameter [nm]	*d*_33_ [pC N^−1^]
Non‐piezoelectric with drawing	14.5 ± 1.7	16 ± 0.3	91.3 ± 10.4	690 ± 110	0
Piezoelectric w/o drawing	56.6 ± 7.6	15 ± 3.7	46.4 ± 6.1	540 ± 120	29.3 ± 2.7
Piezoelectric with drawing	61.8 ± 8.1	31 ± 4.2	39.4 ± 3.2	513 ± 80	36.5 ± 3.9

Concurrently, the stress relaxation/creep of the fibers was significantly reduced (Figure 2D). Thermal‐annealing around the *T*_c_ (90 °C for 1 h) was used to further increase the β‐phase content. The amount of β‐phase content in PDVF‐TrFE samples subjected to post‐synthesis cold‐drawing and thermal annealing was higher than the non‐post‐treated counterparts as determined by FTIR analysis (Figure 2C).

**Figure 3:**
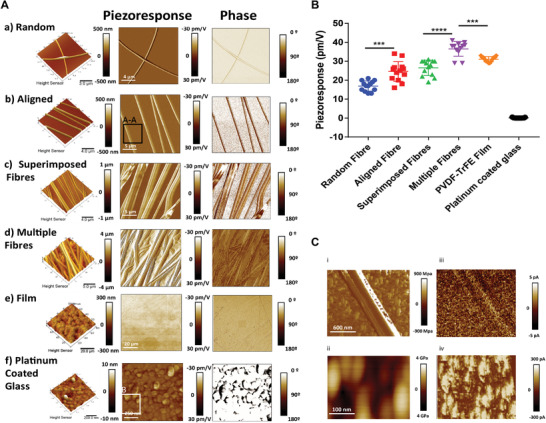
Fibrous aligned piezoelectric scaffolds demonstrated higher piezoelectrical performance than piezoelectric films due to geometrical boundary variables that control the piezoresponse of individual PVDF‐TrFE fibers. PFM amplitude and phase images for single and multiple fibers. A) Random individual fibers (a) demonstrated lower piezoelectric performance relative to aligned individual fibers (b). Aligned superimposed fibers (c) displayed an enhanced piezoelectric response (in‐plane), showing a cooperative effect due to the electromechanical interaction among adjacent fibers. Multiple dense layers (d) of adjacent fibers presented the highest piezoelectric coefficient. Commercial PVDF‐TrFE films (e) display a lower piezoelectric coefficient. A non‐piezoelectric platinum substrate showed no piezoresponse (f). B) Direct comparison of *d*_33_ values (piezoresponse) between samples (a–f). C) Individual fiber DMT elastic modulus (i) and electrical conductivity (iii). Electrical currents (ii) and DMT elastic modulus (iv) were measured on a platinum‐coated glass surface as control, *I* = 300 pA. The relative stiffness difference between single fiber (350 ± 80 MPa) and fiber arrays is responsible for the out‐of‐plane piezoresponse enhancement. No residual (*I* = 0 pA) electrical currents were measured in the individual fibers indicating no resistive mode of conduction characteristic of piezoelectric materials. (All measurements were obtained using *N* = 3 samples, *r* = 7 replicates per sample.) The values are presented as mean ± SD. Significant differences (one‐way ANOVA) of piezoresponse of fibers (*p < 0.05, **p < 0.001) indicate results of the posthoc test (Bonferroni).

The effect of alignment on the piezoelectric performance of individual fibers was demonstrated by switch‐spectroscopy piezoresponse microscopy (SS‐PFM). The piezoelectric response of fibers from randomly‐aligned scaffolds was compared to fibers from mechanically drawn, aligned scaffolds. The piezoresponse or *d*_33_ value was found to be dependent on the fiber alignment. A strong correlation was observed between scaffolds with increased fiber alignment and a higher piezoelectric response (from −16.92 to −24.61 pm V^−1^) (**Figure** **3**). The elastic modulus of individual fibers was 350 ± 80 MPa as measured using peak‐force imaging, yet no significant differences were observed between random and aligned fibers. Interestingly, a cooperative piezoelectric effect previously described by Persano et al. was found in dense arrays of fibers (Figure 3A) due to electromechanical interactions between adjacent fibers and the scaffold stiffness gradient, resulting in an overall enhancement of the scaffold piezoresponse (*d*_33_ = −36.5 ± 3.8 pm V^−1^ and *g*_33_ = −0.41 V m N^−1^).^[^ ^28^ ^]^ Differences between single fibers and multiple fibers (arrays) arise from local differences in stress distribution variations. Discrepancies between obtained values and reported values (*d*_33_ = −29 pC N^−1^) may arise from differences in fiber diameter, orientation, geometry, and arrangement.^[^ ^29^ ^]^

Mechanical and electromechanical stimulation of cells was performed in vitro using non‐piezoelectric PTFE and piezoelectric PVDF‐TrFE scaffolds under cyclic mechanical stretching. The physical and electrical properties of the scaffolds are shown in Table 1.

## 3 The Effect of Electromechanical Stimulation on Tendon‐Specific Gene Expression and Phenotypic Maintenance In Vitro

We investigated the effect of topographical, mechanical, and electrical cues on tendon gene and protein expression. Due to the intrinsic non‐adhesive properties of fluorinated polymers, the fibers were functionalized with an ECM molecule to enhance cell adhesion post‐fabrication.^[^ ^30^ ^]^ Fibronectin functionalization was chosen and characterized (Figure S4, Supporting Information) since it showed higher levels of cell adhesion and triggered a more significant cellular proliferation response compared with collagen type I or PLL coating.^[^ ^31^ ^]^ While fibronectin functionalization might increase integrin‐mediated forces (up to 30 nN^[^ ^10^ ^]^) producing deformations on the piezoelectric fibers and generating interfering electrical signals, using Multiphysics simulations we have found that these level of forces are negligible compared to 4% deformation signals (see Figure S5, Supporting Information) and do not induce any permanent plastic deformations. Therefore, based on these simulations, cell‐adhesion does not change the electromechanical properties of the fibers and threshold for EMS (Figure S5, Supporting Information).

**Figure 4:**
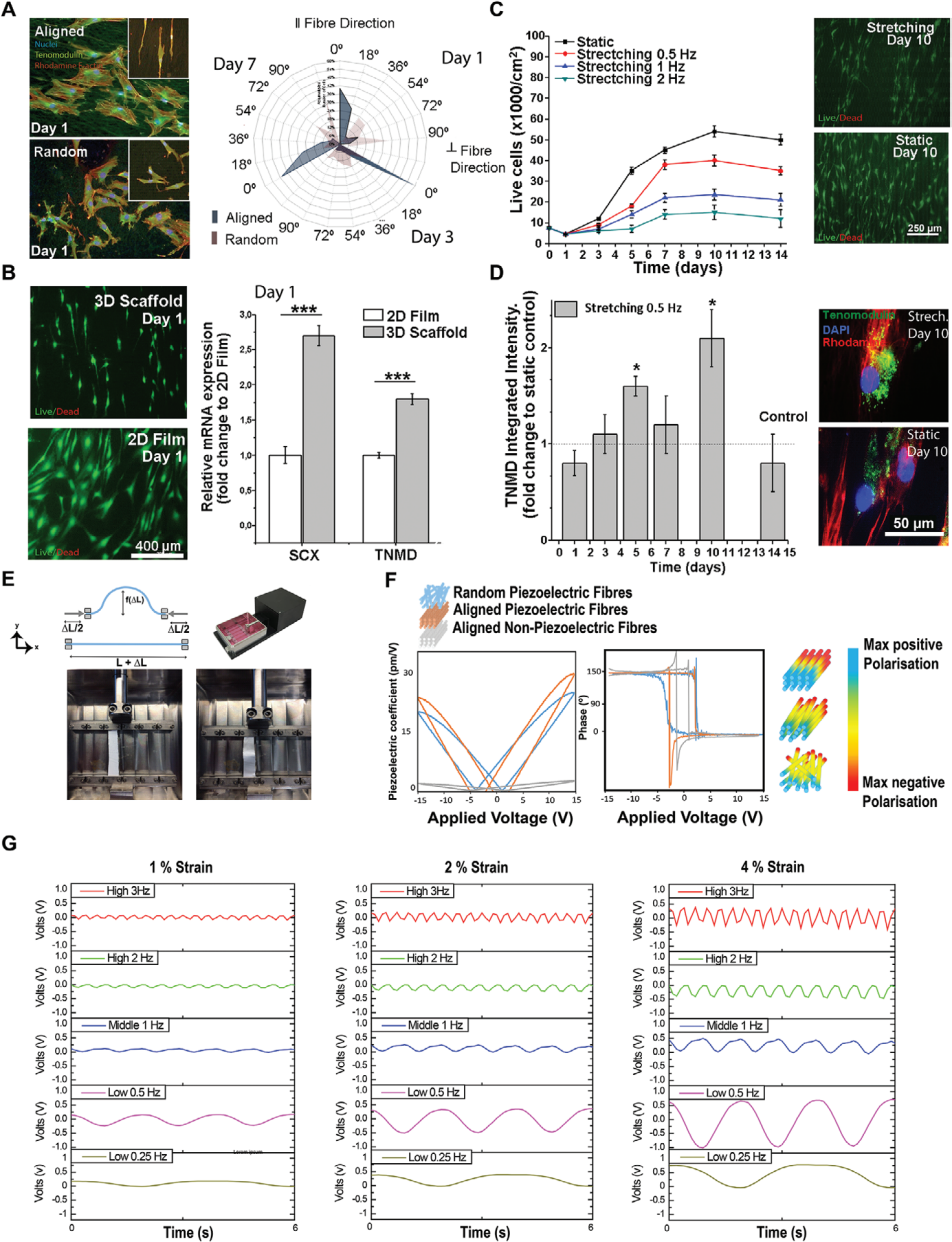
Tenoycte phenotype is promoted by scaffold alignment under specific mechanical loading conditions. A) Human TDCs demonstrated an elongated morphology from day 1 when cultured on aligned PVDF‐TrFE scaffolds. Inserts are higher magnification (red = actin, green = vinculin, and blue = nucleus, scale bar = 400 μm). Angle distribution radar analysis indicated that the percentage of aligned cells was significantly modulated by fiber orientation at day 1, 3, and 7 (0° and 90° correspond to alignment and no alignment, respectively). B) Cells demonstrated differential expression of tenospecific proteins and morphological changes when cultured on electrospun scaffolds or planar PVDF‐TrFE films. hTDCs maintained their phenotype and expression levels of TNMD and SCX when cultured on aligned piezoelectric scaffolds; this effect was absent on 2D planar films (*N* = 3, *r* = 3, mean ± SD, ***p < 0.001). C) hTDCs cultured on piezoelectric scaffolds under dynamic stimulation subjected to different strain rates (0.5, 1, and 2 Hz) exhibited proliferation rates inversely proportional to the strain frequency (*N* = 3, *r* = 3, mean ± SD). D) An increase in the expression of TNMD was observed in cells subjected to 0.5 Hz electromechanical stimulation at days 5 and 10 (*N* = 3, *r* = 3, mean ± SD *p < 0.05). E). An overview of the mechanical loading system used to measure scaffold voltage output under stimulated physiological strain conditions and in vitro analysis. F) Representative hysteresis and butterfly loops of the fiber piezoresponse and polarization. SS‐PFM facilitated the measurement of the PFM phase and amplitude response loops as a function of the applied voltage. The amplitude image shows the magnitude of displacement response generated under a controlled applied voltage in drawn fibers, and the highest amplitude peak was shown to be around 300 pm in response to a 12 V bias. The phase image shows the positive and negative values of antiparallel ferroelectric nanodomains. The phase measurements indicated a ≈180° switch of the dipoles between the applied positive and negative voltages. The piezoresponse obtained from PFM measurements showed that the electrical dipoles lie normal to the surface, characteristic of the organized electrical dipoles/chains of β‐phase crystals. PTFE did not demonstrate piezoelectric behavior (gray line). The schematic indicates the voltage distribution on the fiber surface as a function of geometrical arrangement (fiber orientation) and interaction with adjacent fibers resulting in differing transverse deformation restriction and modulation of the piezoresponse the longitudinal fiber axis. G) Voltage measurements as a function of frequency (0.25, 0.5, 1, 2, and 3 Hz) at a constant amplitude of 1%, 2%, and 4% strain.).

First, we investigated the explicit effect of fiber alignment on cell morphology, cell organization, and expression of the tenocyte‐specific marker Tenomodulin (TNMD) and its transcription factor Scleraxis (SCX) by seeding human TDCs (hTDCs) onto fibrous scaffolds (non‐aligned and aligned) and planar PVDF‐TrFE films for 1, 3, and 7 days (**Figure** **4**). Our observations revealed that early passage (P2) tenocyte cells cultured on planar or non‐aligned fibers presented a polyhedral morphology and ovoid nuclei. Conversely, the fibrous aligned structure promoted increased cell alignment in the longitudinal direction of fibers validated by actin staining. Quantification of alignment over time showed that hTDCs cultured on aligned scaffolds presented cytoskeletal elongation by day 1 and expressed higher levels of SCX and TNMD when compared to cells seeded on both planar films and non‐aligned scaffolds (Figure 4A,B), thus confirming the beneficial effect of fiber‐aligned structures for promoting hTDC phenotype maintenance.

Next, we used a high force uniaxial actuator system to apply specific strain protocols to activate different piezoelectric responses of our devices, previously seeded with hTDCs. Live/dead assay was performed after actuation to demonstrate that the mechanical stretching actuation did not negatively affect the viability of hTDCs in vitro (Figure 4C). To optimize the actuating conditions, we analyzed the piezoelectric performance of the devices, the hTDC viability, the nuclear deformation, and the TNMD and SCX expression (Figure 4C,D; Figure S6, Supporting Information) under stretching conditions. As piezoelectric systems exhibit frequency‐modulated performance (output charge) due to the frequency dependence of the elastic/piezoelectric constants, we assessed the effect of strain rate (0.25, 0.5, 1, 2, and 3 Hz) on the piezoelectric constant *d*_31_ using physiologically relevant strain magnitudes (1%, 2%, and 4%) (Figure 4E,G). In general, aligned PVDF‐TrFE fibers produced a significantly increased piezoelectric performance when the application of force was parallel to the fiber orientation (Figure 4F). As anticipated, voltage measurements showed a positive correlation between voltage output and strain magnitude, demonstrating the device's piezoelectric nature (also confirmed using SS‐PFM, Figure 4F). At a fixed frequency (i.e., 0.25 Hz) the voltage output increased linearly with increasing strain magnitude (from 0.28 V at 1% to 1.21 V at 4%, respectively). At fixed strain magnitude (i.e., 4%), the voltage output ranged from 0.51 V at 3 Hz to 1.21 V at 0.25 Hz (Figure 4G). We then tested the voltage output stability of the device up to 500 cycles of continuous dynamic strain, and no significant change to the device voltage output (Figure 2D) was observed. Finally, we chose 0.5 Hz frequency and 4% strain magnitude for subsequent genomic analyses (**Table** **2**) as cell viability was preserved (comparable to tissue culture plate, Figure 4C, *p* > 0.05 for all days). As shown in our cell viability data (Figure S7, Supporting Information), we have observed a transient effect in cell viability at day 7 in dynamic (4% strain, 0,5Hz, 8 h per day) versus static conditions. Importantly, this effect was not material/treatment dependent as the cell viability was identical for piezoelectric and non‐piezoelectric scaffold in both static and dynamic conditions. At the same time, the TNMD expression was significantly increased (fold‐increase of ≈1.5 and ≈2, after 5 and 10 days of stimulation respectively, Figure 4D). Scaffolds demonstrate a high level of piezoelectric output (1 V), illustrating the potential of our actuated piezoelectric devices (electromechanical stimulation) in sustaining hTDC proliferation and promoting a tendon‐like phenotype.

**Table 2:** QIAGEN genes list for customized gene array

Symbol	Entrez gene name
ABCB1	ATP binding cassette subfamily B member 1
ACAN	Aggrecan
ACTA1	actin, alpha 1, skeletal muscle
ACVR1	activin A receptor type 1
AHSG	alpha 2‐HS glycoprotein
ALPL	alkaline phosphatase, liver/bone/kidney
BGLAP	bone gamma‐carboxyglutamate protein
BGN	Biglycan
BMP1	bone morphogenetic protein 1
BMP2	bone morphogenetic protein 2
BMP4	bone morphogenetic protein 4
BMP6	bone morphogenetic protein 6
BMP7	bone morphogenetic protein 7
BMPR1A	bone morphogenetic protein receptor type 1A
BMPR2	bone morphogenetic protein receptor type 2
CASP3	caspase 3
COL11A1	collagen type XI alpha 1 chain
COL14A1	collagen type XIV alpha 1 chain
COL1A1	collagen type I alpha 1 chain
COL1A2	collagen type I alpha 2 chain
COL2A1	collagen type II alpha 1 chain
COL3A1	collagen type III alpha 1 chain
COL4A1	collagen type IV alpha 1 chain
COL5A1	collagen type V alpha 1 chain
COL6A1	collagen type VI alpha 1 chain
COMP	cartilage oligomeric matrix protein
DCN	Decorin
DLX5	distal‐less homeobox 5
EGR1	early growth response 1
FGF10	fibroblast growth factor 10
GDF15	growth differentiation factor 15
GDF5	growth differentiation factor 5
GDF6	growth differentiation factor 6
GDF7	growth differentiation factor 7
HAT1	histone acetyltransferase 1
HDAC1	histone deacetylase 1
HNF1A	HNF1 homeobox A
IBSP	integrin binding sialoprotein
IGF1	insulin like growth factor 1
ITGA1	integrin subunit alpha 1
ITGA2	integrin subunit alpha 2
ITGA3	integrin subunit alpha 3
ITGA4	integrin subunit alpha 4
ITGA5	integrin subunit alpha 5
ITGAX	integrin subunit alpha X
ITGB1	integrin subunit beta 1
ITGB3	integrin subunit beta 3
ITGB5	integrin subunit beta 5
KAT2B	lysine acetyltransferase 2B
KCNK2	potassium two pore domain channel subfamily K member 2
KCNK4	potassium two pore domain channel subfamily K member 4
KDR	kinase insert domain receptor
MGP	matrix Gla protein
MKX	mohawk homeobox
PIEZO1	piezo type mechanosensitive ion channel component 1
PIEZO2	piezo type mechanosensitive ion channel component 2
PIK3CG	phosphatidylinositol‐4,5‐bisphosphate 3‐kinase catalytic subunit gamma
PTEN	phosphatase and tensin homolog
PTK2	protein tyrosine kinase 2
PTK2	protein tyrosine kinase 2
PXN	Paxillin
RUNX2	runt related transcription factor 2
SCX	scleraxis bHLH transcription factor
SMAD3	SMAD family member 3
SMAD4	SMAD family member 4
SMAD9	SMAD family member 9
SMURF1	SMAD specific E3 ubiquitin protein ligase 1
SMURF2	SMAD specific E3 ubiquitin protein ligase 2
SOX9	SRY‐box 9
SP7	Sp7 transcription factor
SPARC	secreted protein acidic and cysteine rich
SPP1	secreted phosphoprotein 1
TBX5	T‐box 5
TGFB1	transforming growth factor beta 1
THBS4	thrombospondin 4
TLN1	talin 1
TNC	tenascin C
TNMD	Tenomodulin
TRPA1	transient receptor potential cation channel subfamily A member 1
TRPV1	transient receptor potential cation channel subfamily V member 1
TWIST1	twist family bHLH transcription factor 1
VCL	Vinculin
VEGFA	vascular endothelial growth factor A
ZYX	Zyxin

**Figure 5:**
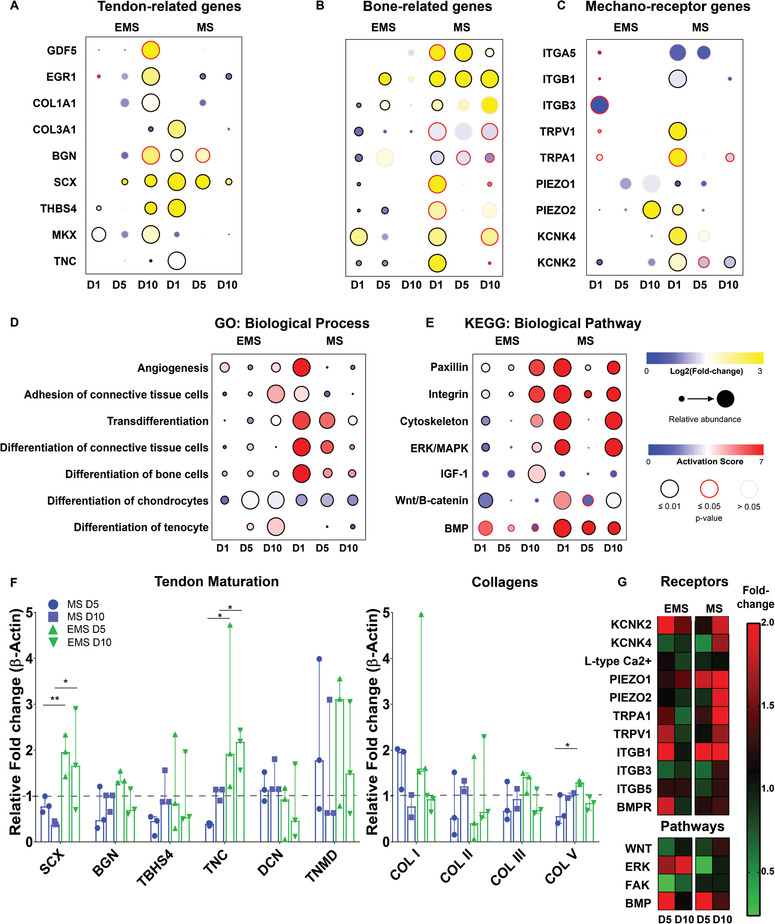
Electromechanical stimulation induces the maintenance of tendon specific gene expression and phenotype in vitro. Dot plots represent gene expression of human hTDCs cultured under mechanical or electromechanical stimulation for 1, 5 and 10 days. A) Expression of genes related to tenogenesis, B) bone formation and C) mechanoreceptors (*N* = 3, *r* = 6). The significant changes in gene expression were classified into D) specific biological processes and E) signaling pathways. In particular, electromechanical stimulation for 10 days induced a significant activation of tendon specific differentiation pathways associated with the activation of MAPK and activity‐dependent downregulation of TRPA1 and Piezo1&2 ion channels. This trend was reversed in cells exposed to mechanical stimulation alone. F) Expression profiles of tendon specific proteins and collagens in hTDCs subjected to electromechanical (EMS) and mechanical (MS) stimulation at day 5 (D5) and day 10 (D10). A significant increase in Col. V synthesis was observed during EMS relative to MS at day 5 (median ± ra; *p < 0.1, **p < 0.01). G) Heatmap of ion channel, integrins, and tendon/osteochondral specific protein expression in hTDCs subjected to EMS and MS at day 5 and 10 (median ± range; *N* = 4, *r* = 8). Non‐parametric Kruskal–Wallis followed by Dunn's multiple‐comparisons test was used.

Tendons have a great ability to respond to mechanical forces by adapting their structure and biochemical composition and cyclic mechanical stretching of the tendon is a recognized method for treating tendon‐related injuries.^[^ ^32^ ^]^ Several studies have confirmed that tendon is subjected to 3–4% strain during normal activities^[^ ^33^ ^]^ and, that strain rate (loading frequency) is important in modulating the cellular response in vitro.^[^ ^34^ ^]^ Under physiological conditions, the strain rate of the tendon is around 0.1–0.5 Hz; however, during intense activity, the frequency can be as high as 10 Hz.^[^ ^35^, ^36^ ^]^ Recently, it has been demonstrated that tendon cells modulate their gene expression, protein synthesis and mitogenesis in vitro through activation of mechanotransductive signaling pathways under physiological mechanical stimulation.^[^ ^37^ ^]^ Therefore, we compared the effect of mechanical and electromechanical stimulation on human hTDCs gene and protein expression (**Table** **3**, **4** and **5**) (**Figure** **5**). Genomic analysis of human hTDCs cultured on piezoelectric PVDF‐TrFE and non‐piezoelectric PTFE scaffolds under both static (4% static strain) and physiologically relevant dynamic loading conditions (4% dynamic strain at 0.5 Hz for 8 h per day) was performed at 1, 5, and 10 days. Overall, the analysis of gene expression correlated well with the results observed at the protein level (Figure 5D). Electromechanical stimulation (EMS) using actuated PVDF‐TrFE scaffolds, induced a rapid and sustained up‐regulation of tendon‐related and bone‐related genes relative to static controls at day 1. Interestingly, after 10 days of EMS bone‐related genes returned to basal levels whereas tendon‐related genes remained upregulated, suggesting a strong effect of EMS toward tenogenic differentiation. Similarly, mechanical stimulation alone (MS) through PTFE scaffolds induced a significant up‐regulation of bone and tendon‐related genes at day 1 (relative to static counterpart), but unlike EMS conditions, bone‐related genes remained up‐regulated while tendon‐related genes returned to control levels by days 5 and 10 (Figure 5A,B; Figure S8, Supporting Information). Specifically, by day 10, bone‐related genes were up‐regulated in hTDCs subjected to mechanical stimulation (Figure 5A,B; Figure S8, Supporting Information), whereas SCX and TNMD were upregulated only in the EMS group (Figure 5E). Taken together, these observations indicate that trans‐differentiation of hTDCs toward an osteogenic/chondrogenic lineage can be modulated by EMS and MS. Moreover, EMS offered sustained tenogenic differentiation capacity relative to MS alone.

**Table 3:** Tenogenesis array: proteins associated with tendon regeneration or hTDC function

Proteins	Abbreviation	Purchased from	Cat. no
Scleraxis	SCX	Abcam	ab58655
Tenomodulin	TNMD	Abcam	ab203676
Byglican	BGN	Abcam	ab49701
Decorin	DCN	Abcam	ab175404
Thrombospondin 4	THBS‐4	Abcam	ab176116
Tenascin C	TNC	Abcam	ab88280
Collagen I	COLI	Abcam	ab138492
Collagen II	COLI	Abcam	ab185430
Collagen III	COLIII	Abcam	ab7778
Collagen V	COLV	Abcam	ab7046

**Table 4:** Signaling array: proteins associate to different signaling pathways (MAPK, FAK, TGF‐B, BMP, and WNT)

Proteins	Abbreviation	Purchased from	Cat. no
Smad 1	SMAD1	Cell Signaling	6944S 6944S
Smad 5	SMAD3	Cell Signaling	12534S
Phosphorylated Smad 1	SMAD1/5/8	Cell Signaling	5753S
Phosphorylated Smad 1/5/8	pSMAD158	Cell Signaling	9516s 9516s
Focal adhesion kinase	FAK	MBL	D061‐3 12G4
Phosphorylated FAK	pFAK	Cell Signaling	3284S 3284S
MAPK	ERK	Cell Signaling	4696S L34F12
p44/42 MAPK	pERK	Cell Signaling	4377S
Wnt/β‐catenin	β‐Catenin	Millipore	2 858 901
Active β‐catenin, clone 8E7	Active β‐Catenin	Millipore	ABC
β‐actin	β‐actin	WAKO	019‐19741

**Table 5:** Receptors array: proteins associate to cell membrane receptors

Proteins	Abbreviation	Purchased from	Cat. no
TRPV1	TRPV1	SantaCruz	sc‐20813
Piezo1	Piezo1	SantaCruz	sc‐164319
Piezo2	Piezo2	SantaCruz	sc‐84763
TRPA1	TRPA1	SantaCruz	sc‐32353
KCNK2	KCNK2	SantaCruz	sc‐11557
KCNK4	KCNK4	Abcam	ab81367
L‐type Ca^2+^	L‐type Ca^2+^	SantaCruz	sc‐25686
BMPR1A	BMPR1A	ThermoFisher	PA5‐11856
Integrin1	ITG1	Abcam	ab134179
Integrin3	ITG3	Abcam	ab34409
Integrin5	ITG5	Cell Signaling	3629S

To unravel the signal transduction pathways associated with the regulation of tenogenic differentiation, ingenuity pathway analysis (IPA) was used with gene expression profiles of hTDC exposed to MS and EMS stimulation. IPA attributes an activation *z*‐score used to predict the activation state of biological functions and signaling pathways. Pathway analysis of human tendon derived cells exposed to either EMS or MS yielded significant modulation of broad functional signaling pathways, and a mechanistic network of genes and specific biological functions were differentially regulated (Figure S9, Supporting Information). Canonical signaling pathways with the most significant number of modulated gene expression were associated with day 1 and 10 in vitro (Figure S9, Supporting Information). The ERK/MAPK signaling pathway was consistently activated with both EMS and MS stimulation; however, this pathway was significantly upregulated in hTDCs subjected to MS at days 1 and 10 (Figure 5E).

Similarly, increased activation of cell transdifferentiation and differentiation toward the bone cell lineage and upregulations in osteogenic signaling pathways Wnt/β‐Catenin and BMP were attributed to MS at day 10 (relative to EMS). Significantly, following 10 days in culture, the tendon‐related signaling pathway IGF‐1 and biological differentiation process associated with tenocyte function were activated in tendon derived cells subjected to EMS but downregulated under MS (Figure 5E). We looked at the protein expression profile of tendon‐specific maturation markers and collagen synthesis to validate gene expression results. In agreement with the gene expression data, we observed a higher expression of tendon maturation markers (SCX and TNC) of tendon derived cells exposed to EMS relative to MS in addition to the increased synthesis of collagen type V (Figure 5F,G).

During tendon specific differentiation or trans‐differentiation, highly regulated signal transduction events take place, leading to the expression of genes associated with the tendon‐specific phenotype or phenotypic drift respectively. We hypothesized that mechanically activated and electrically gated membrane ion channels would undergo modulated expression in response to EMS. To investigate the role of these membrane sensors on signal generation and propagation, correlational analysis was conducted between significant modulations to tendon‐specific functional pathways at day 1, 5, and 10 in the mechanistic network to differential changes in the expression of mechano‐sensitive (TRP family ion channels, focal adhesion‐related proteins and integrins β1, β3, and β5), electro‐sensitive (KCNK family and Ca^2+^ L‐type ion channel), and piezo‐sensitive (Piezo family ion channels) receptors, as well as the BMP receptor BMPR1A^[^ ^38^, ^39^ ^]^ (Figure 5C). Critically, increased activation of tendon differentiation‐related genes (Figure 5A) through the modulated activity of tendon‐related transcription factors (i.e., SCX and EGR1, see Figure S8, Supporting Information) was correlated with significant modulation of the expression level of TRP family ion channels under stimulation (Figure 5G). As shown in Figure 5F, the expression of SCX and TNC was significantly increased under EMS conditions at both day 5 and 10, together with a significantly lower expression of KCNK4, TRPA1, and TRPV1 ion channels when compared to the MS group. These results were further validated at the protein level (Figure 5G).

Conversely under MS, a decrease in the expression of tendon‐related genes and an increase in the expression of genes associated with cartilage/bone differentiation was associated with higher expression of TRP ion channels (Figure 5E,G; Figure S8, Supporting Information). Specifically, TRPA1 and TRPV1 ion channels underwent a sustained increase in expression which correlated with a positive regulation of transcription factors SOX9, RUNX2, and COMP, proteins that have a recognized role in the processes of osteogenesis (Figure 5B; Figure S6, Supporting Information). Overall, the gene expression results and pathways analyses, further validated using a custom‐made protein array, demonstrated that MS of hTDCs in vitro resulted in a significant increase in the expression (sensitivity) of ion channels (PIEZO1, PIEZO2, KCNK4, TRPA1, TRPV1) and activation of BMP signaling whereas EMS resulted in a modulation of ion channels and deactivation of BMP (Figure 5E,G).

## 4 Electromechanical Stimulation Promotes the Activation of Tendon Specific Signaling Pathways In Vivo

**Figure 6:**
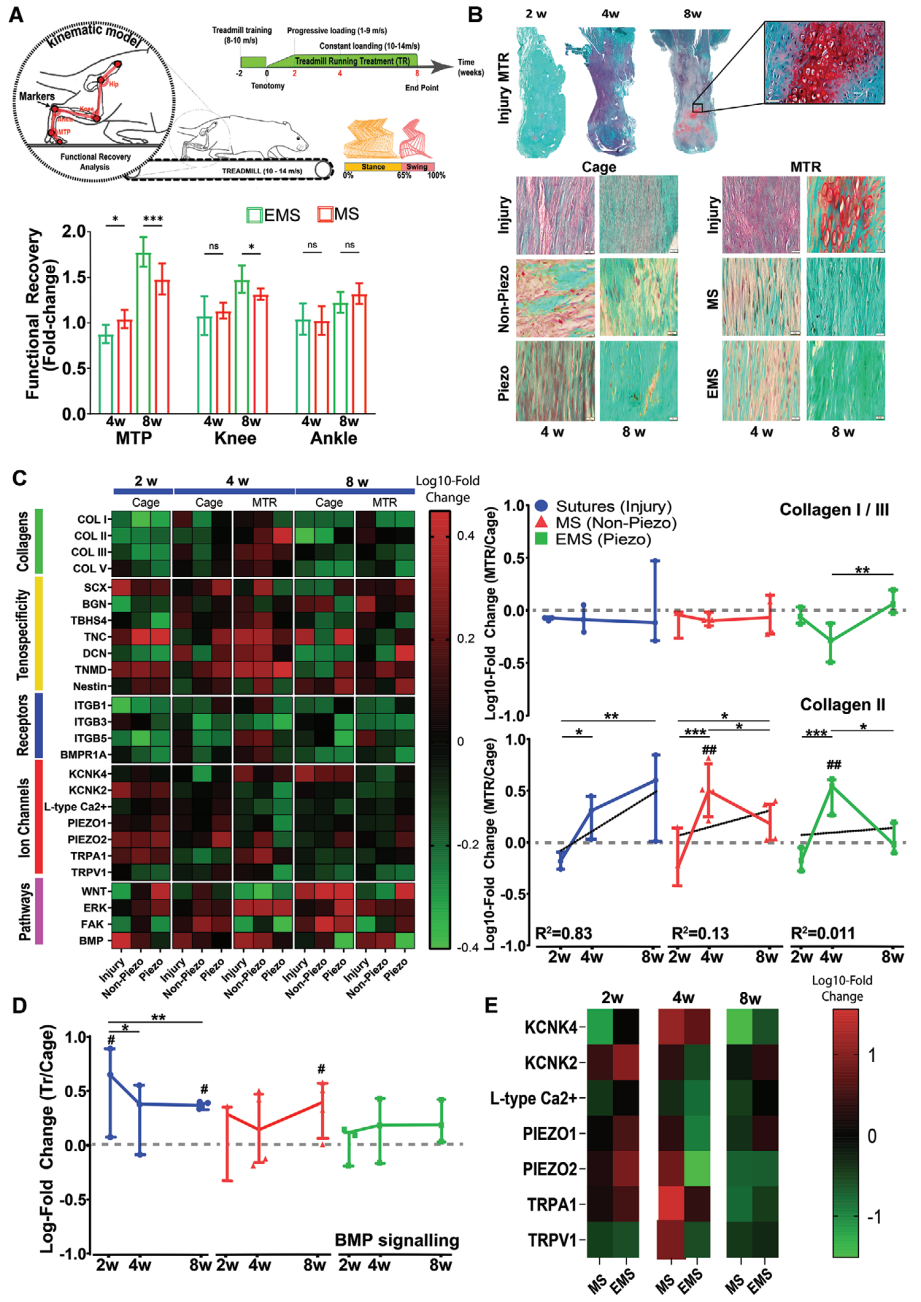
Electromechanical stimulation promotes collagen synthesis and controls tendon‐specific protein expression in vivo. A) Schematic representation of the kinematic model used to assess the functional recovery of tendon based on gait analyses and the moderate treadmill running treatment protocol timeline. The graph shows the Fold‐change of the functional recovery between EMS (Piezo + MTR) and MS (Non‐Piezo + MTR) for MTP, knee, and ankle. B) Representative histological images of Safranin‐O stained samples for MTR‐treated and caged groups. The progression of calcification is observed in the Injury (Cage and MTR) group. C) Overview of the microarray heatmap depicting the log10‐fold change of protein expression for each group (treatment vs contralateral) related to collagens synthesis, tendonspecificity, cellular receptors, ion channels, and specific pathway activation. Data represent the median value of fold change relative to the control sample (contralateral intact tendon) and using β‐actin as loading control. D). Fold‐change in the activation state of BMP signaling shows consistent activation for MS and Injury after 8 wpi. E) Heatmap depicting the fold change of mechanosensitive ion channels expression between MS and EMS. Data is represented as median ± IQR (*N* = 4, *r* = 8). Non‐parametric Kruskal–Wallis followed by Dunn's multiple‐comparisons test was performed. *, ** represents the statically significant difference between MTR groups (*p* < 0.05) and (*p* < 0.01), respectively. #, ## represents the statically significant difference of a MTR group with respect to control caged group (*p* < 0.05) and (*p* < 0.01), respectively.

Finally, we investigated the influence of MS and EMS at the tissue level, where transient mechanical cues can alter cellular function and subsequent changes in matrix composition and architecture to maintain tendon homeostasis.^[^ ^40^ ^]^ We used a full‐thickness Rat Achilles acute tendon injury model where 5 mm of tendon was removed and a standard moderate treadmill running protocol (MTR) to impact tissue‐repair processes mechanically and consistently induce a piezoelectric response from the devices (**Figure** **6**; Figures S10–S14, Supporting Information). Critically, it was noted that cells physically distant from the scaffold may also be affected by electromechanical stimulation (EMS). As shown by the computational modeling, EMS affects a significant larger peri‐implant tissue volume, potentially contributing more to tissue formation and regeneration than topographical cues that are only sensed locally by the cells adhered to the device surface (see Figures S11 and S12, Supporting Information).

Tendon injuries were repaired by suturing the tendon end to end and either placing a scaffold (non‐piezoelectric or piezoelectric) in the 5 mm defect or maintaining the defect without a supporting scaffold (Injury alone); Figure S10, Supporting Information). 2‐week post‐injury (wpi) (inflammatory phase) animals were subjected to daily MTR activity or remained under cage roaming conditions (Cage); Table S4, Supporting Information). Consistent with previous studies, MTR resulted in the enhanced function of repaired tendons regardless of the treatment. After 8 wpi, MTR groups (Injury MTR, Non‐Piezo MTR, and Piezo MTR) displayed a significant increase in their functional recovery (together with histological changes) in comparison to their respective caged groups (Injury Cage, Non‐Piezo Cage, and Piezo Cage, *p* < 0.05, *p* < 0.05, and *p* < 0.05, respectively) demonstrating the tissue repair regulatory effect of mechanical cues (Figure 6A and Table S4, Supporting Information). By directly comparing tissues repaired under MTR and Cage conditions (Piezo MTR relative to Piezo Cage and Non‐Piezo MTR relative to Non‐Piezo Cage) we explored the histological differences between EMS and MS, respectively. Significant increases in tissue organization (Figure 6A and Table S4, Supporting Information) assessed by level of fiber orientation (quantified by polarized microscopy in picrosirius stained samples, Figure S15, Supporting Information), and a decrease in calcifications assessed by presence of glycosaminoglycans (GAGs) (quantified by Safranin‐O staining intensity; Figure 6B; Figure S16, Supporting Information) were observed in tendon tissues derived from animals which were treated with EMS compared to MS (Figures S17 and S18, Supporting Information). Specifically, we measured peri‐implant cell number, morphology, and alignment at different locations (proximal, middle, and distal) during mechanical and electro‐mechanical stimulation in vivo. As shown in the Figures S14 and S18, Supporting Information, a calcification response was noted in the distal area (connection between the scaffold and bone) as observed in previous studies.^[^ ^41^ ^]^ Critically, this response was more pronounced in cells exposed only to mechanical stresses which underwent osteochondral transdifferentiation as shown by an increase in GAGs staining intensity and distribution, an increase in cell number and loss of elongated morphology.

In intact healthy tendons (sham) cells normally show an elongated and organized morphology (Figure S9b,c, Supporting Information), and reside in a rich and dense ECM composed of aligned collagen type I cross‐linked by low amounts of GAGs (Figures S17 and S18, Supporting Information). In healed tissues using clinical standard methodology of end to end suturing (injury) and under free‐roaming conditions (cage), resident and invading cells adopted a non‐elongated morphology and random organization and after 8 weeks post‐injury, demonstrated regions of calcification (Figure 6B; Figure S11, Supporting Information). In comparison, scaffold‐mediated tendon repair (Non‐Piezo and Piezo) reduced this calcification (Figure 6B and Table S5, Supporting Information) considerably and was associated with enhanced tissue organization and decrease in BMP signaling activation (Figure S19, Supporting Information).

Finally, we used a customized protein array to evaluate collagens synthesis, tenospecificty, cell mechanoreceptor expression, ion channels, and signaling pathways activation (Figure 6C and Tables 3, 4 and 5). We found that collagen I/III synthesis and tendon specific proteins were significantly increased upon EMS at 8 wpi (with respect to 4 wpi). Unlike tissues under MS, cartilage‐marker collagen II decreased in tissues under EMS, demonstrating the potential of electrical cues on regulating pathological tissue processes (Figure 6C). Protein microarray analysis revealed that SCX and TNMD were significantly upregulated at 2 wpi and differentially regulated at 4 and 8 wpi under EMS. Compared to MS, SCX, and TNMD expressions demonstrated significant upregulations (>twofold change) during tendon healing under EMS after 8 wpi (Figure 6D). These findings were consistent with the results obtained with our in vitro model; specifically, MS (Injury TR and Non‐Piezo TR) resulted in sustained activation of BMP signaling pathway (2, 4, and 8 wpi, Figure 6D).^[^ ^42^ ^]^

Based on previous studies, it is known ion channel modulation occurs immediately after injury, initiating signaling pathways such as calcium and the mitogen activated protein kinase (MAPK) extracellular signal‐regulated kinase (ERK), which are essential for successful tissue repair.^[^ ^43^, ^44^, ^45^ ^]^ However, it is well recognized that injury signals often result on activation of unspecific repair processes. In our in vivo model, injury stimulus resulted in early increase in SCX (Injury, *p* < 0.01) and TNMD (Injury, *p* < 0.05) expression at 2 wpi together with high levels of expression of KCNK2 (Injury, *p* < 0.01), TRPA1 (Injury, *p* < 0.05) and PIEZO2 (Injury, *p* < 0.05) (Figure 6C). As tissue repair progressed into the tissue remodeling processes (4 and 8 wpi), a decrease in tenospecificity and increase in the expression of collagen II occurred (Injury MTR, *p* < 0.05; non‐piezo, *p* < 0.05 at 4 wpi, and Injury MTR, *p* < 0.05 at 8 wpi) in conjunction with elevated expression of BMPR (Non‐Piezo TR, *p* < 0.001 at 4 wpi) and a sustained activation of BMP signaling pathway (Injury, *p* < 0.05 and Non‐Piezo, *p* < 0.05 at 2, 4, and 8 wpi) (Figure 6C,D).

Notably, the sustained and prolonged activation of BMP signaling was associated with MS but was not present with EMS. For MS, BMP signaling started at 2 wpi, remained activated at 4 wpi and peaked at 8 wpi for both Injury MTR (*p* < 0.05) and Non‐Piezo MTR groups (*p* < 0.05) (Figure 6D). In turn, ion channel expression was high at 2 and 4 wpi but reduced at 8 wpi within the MS group (Figure 6E). In particular, the ion channels modulated by MS were TRPA1 (Injury MTR, *p* < 0.01 and Non‐Piezo MTR, *p* < 0.05 at 2 and 4 wpi), KCNK2 (Injury MTR, *p* < 0.01 and Non‐Piezo MTR, *p* < 0.05 at 2 wpi) and PIEZO2 (Injury MTR, *p* < 0.05 and Non‐Piezo MTR at 2 and 4 wpi). Relative to MS, the favorable regulatory effect of EMS at 8 wpi was associated with low levels of BMP signaling and low expression of ion channels KCNK2 (*p* < 0.05), PIEZO1 (*p* < 0.05), PIEZO2 (*p* < 0.05) and L‐type Ca^2+^(*p* < 0.05) at 4 wpi but relatively high at weeks 2 and 8 wpi. Taken together these results demonstrate that EMS affects tendon‐specific tissue repair processes by limiting the activation of the BMP signaling pathway through temporal modulation of mechanosensitive ion channels expression.

## 5 Discussion

Previous studies have demonstrated that tendon matrix properties such as fiber alignment, anisotropic mechanical properties and electrical charge influence tissue repair and cellular function, yet the combinational roles of these properties have not so far been elucidated.^[^ ^16^, ^46^, ^47^, ^48^, ^49^, ^50^ ^]^ Here we developed a piezo‐bioelectric device capable of cellular interfacing and demonstrated that electromechanical stimulation influences tendon tissue‐repair signaling pathways by modulating mechanosensitive ion channels expression. Furthermore, by fabricating a (chemically and topographically analogous) non‐piezoelectric device, we demonstrated a more significant contribution from bioelectric cues in promoting tendon specific signaling pathways than mechanical cues. Electrospinning of PVDF‐TrFE always results in a high content of the polar phase (β‐phase) due to the steric hindrance of TrFE in the copolymer, associated extensional forces as well as to the exposure to strong electric fields causing local poling. To the best of our knowledge and ability, and unlike PVDF‐TrFE films, electrospun scaffolds cannot be obtained entirely in the non‐polar (α‐phase) form. Studies have shown that modulating piezoelectricity in electrospun scaffolds is possible by using thermal treatments (above *T*_c_, >110 °C).^[^ ^1^, ^2^ ^]^ However, this inevitably results in a loss of aligned fibrous structure and elasticity and only allows for the generation of relatively low‐level piezoelectric scaffolds, not for the generation of electrically inert scaffolds. For our study, we chose to fabricate an entirely electrically inert scaffold from PTFE as a control against a highly piezoelectric scaffold fabricated using a low annealing temperature (90 °C) and a cold drawing process. In this way, the aligned structure, elasticity, and morphology were comparable between experimental and control scaffolds. Most previous studies have compared the effects of electrospun PVDF‐TrFE to commonly used biopolymers in tendon tissue engineering, such as polycaprolactone (PCL) or polylactic acid (PLA), which are employed as non‐piezoelectric controls; however, in these cases, the chemistry is notably different, and the results cannot solely be ascribed to piezoelectricity alone. Furthermore, biopolymer scaffolds such as collagen are also inappropriate as a control owing to the inherent piezoelectric properties of the fibrillar form.^[^ ^3^ ^]^ In contrast, we have opted to fabricate an entirely chemically and physically analogue electrospun non‐piezoelectric alternative (electrospun PTFE). This is the first time the biological effects of piezoelectricity alone are shown by comparing piezoelectric and chemically analogue non‐piezoelectric electrospun scaffolds. Finally, we demonstrated the potential of electromechanical stimulation in regulating tendon tissue repair processes and associated‐signaling pathways in vivo.

The expression of Tnmd has been shown to become upregulated in tendon cells when cultured on the aligned scaffold and mechanically stimulated.^[^ ^51^ ^]^ Our results were consistent with these findings, where hTDCs cultured on aligned fibrous scaffold demonstrated a typical tenocyte‐like morphology together with high levels of Tnmd. Conversely, hTDCs cultured on planar control surfaces showed low Tnmd mRNA levels and phenotypic drift (transdifferentiation). Upon mechanical stimulation (4% strain, 0.5Hz, 8 h per day), the expression of Tnmd in hTDCs was further upregulated along with the Tnmd transcription factor Scx and enhanced collagen I synthesis. It is well established that tendon cells can sense and transduce environmental mechanical cues into activation of downstream signaling pathways to regulate their function and participate in tissue‐level repair processes. Studies have found that calcium activity and endogenous electrical fields associated with tissue injury re‐activate signaling pathways involved in embryonic development to promote tissue regeneration. These studies have noted that upon tissue damage, injury‐related electrical potentials are created and result in direct current‐like stimulation (1–2 V cm^−1^) that decays over time.^[^ ^52^, ^53^ ^]^ Thus, we hypothesized that natural healing events could be recapitulated using a piezo‐bioelectric device made of PVDF‐TrFE fibers that recreates the modulated electrical polarization and calcium activity occurring after injury and can interact with specific regenerative pathways to promote enhanced regeneration. Although the role of piezoelectricity on tissue regeneration remains elusive, considerable studies have demonstrated the potential of electrospun PDVF‐TrFE for modulating cellular responses within the context of tissue engineering.^[^ ^17^, ^18^, ^19^, ^20^ ^]^ In a recent study, Sita et al. fabricated a PVDF‐TrFE fibrous scaffold capable of generating enough electrical outputs to control hMSC differentiation and tissue formation in vitro under physiological loading conditions. This study demonstrated the significant additive effect of bioelectric cues alone by showing electric field‐cellular differentiation. Here, lower voltages promoted chondrogenic differentiation, and higher voltages resulted in osteogenesis and have become increasingly clear that electric fields affect differentiation processes. Furthermore, the highly aligned fibrous architecture inherent to specific biological tissues is also recognized as critical in mediating biological functions. For instance, Lee et al. showed that aligned electrospun PVDF‐TrFE fibers coated with collagen promoted DRG neurite outgrowth compared to random fibers in vitro^[^ ^19^ ^]^ and in vivo.^[^ ^55^ ^]^

Based on our bioinformatic analysis of gene expression of human tendon cells exposed to MS or EMS, we identified MAPK, FAK, Wnt/β‐Catenin, and BMP as the most differentially regulated signaling pathways. Previous studies have found that following tendon injury, excessive mechanical stimulation can lead to concurrent activation of tenospecific signaling pathways and osteogenic events through BMP signaling pathways, resulting in ectopic calcifications perturbating the tendon‐specific biomechanical properties. Our results showed that MS resulted in significant and sustained BMP signaling, high expression of ion channels, and loss of tendon specificity in tenocyte populations in vitro. Conversely, EMS resulted in the maintenance of tendon phenotype (shown by the upregulation of Tnmd and Scx expression) and modulated BMP signaling processes.

A subsequent experiment with a full‐thickness Achilles tendon rat injury model supported our in vitro findings, where mechanical stimulation through a moderate treadmill running protocol enhanced unspecific tissue repair processes, including ectopic bone formation in vivo. Indeed, after 2 wpi, we observed that the activation of BMP signaling occurred in response to the acute injury and increased the synthesis of collagen II (a marker associated with osteochondral formation) at 4 and 8 wpi in animals exposed to MS. Conversely, the expression of markers of tendon maturation SCX, TNMD increased under EMS but not under MS, at 8 wpi as associated with increased collagen I/III ratio. It is worth noting that BMP signaling after 8 weeks post injury was significantly reduced (compared to MS and sutures; Figure 6D, *p* < 0.05) while mechanosensitive ion channel expression was highly modulated in animals exposed to EMS. Admittedly, the exact underlying mechanism for tissue specificity and interaction between calcium and BMP signaling remains elusive and should be further investigated.

## 6 Conclusion

This work shows that tendon cell biological function and tissue repair processes can be modulated by mechanical and electromechanical stresses. We improved the therapeutic performance of the implantable devices by enhancing the physicomechanical, piezoelectric, and biological characteristics through a short‐distance electrospinning process followed by fibronectin functionalization. In vitro stimulation of hTDCs with mechanical and bioelectric cues revealed that BMP, MAPK, FAK, and Wnt/β‐catenine form a complex signaling network that controls tenogenic cell differentiation. Furthermore, we evaluated the individual role of mechanical, topographical, and electrical cues and showed that bioelectric cues contribute significantly in promoting a tendon specific phenotype characterized by changes in gene and protein expression, biological functions, pathway analyses, and cell morphology. Finally, using a mechanically‐loaded acute tendon injury rat model to mimic the profile of tendinopathy, including calcification, we demonstrated the efficacy electromechanical stimulation (EMS) in modulating the expression of ion channels (TRPA1, PIEZO1/2, and KCNK2/4) and in regulating perturbed regenerative processes associated with excessive activation of the BMP signaling. EMS was enabled by the use of an implantable piezo‐bioelectric device during post‐injury mechanotherpy and represents a paradigm shift in the management of severe tendon injuries or calcific tendinopathy without the use of drugs or external stimulation. Importantly, this study establishes the engineering foundations for a broad range of synthetic piezo‐bioelectric scaffolds that enable bioelectric control of specific tissue regenerative processes.

## 7 Experimental Section

### Cell Culture

Healthy human tendon derived cells (hTDCs) were isolated from Achilles tendon (2 males of 63 and 66 years old), patellar tendon (1 male 60 years old), peroneal tendon (1 male 55 years old) and extensor digitorum (two females of 28 and 13 years old) tendons during tendon grafting operations after obtaining written informed consent, ethical approval and licenses in the University Hospital Galway (C.A. 1046). Cells were isolated using the migration method, as previously described.^[^ ^56^ ^]^ hTDCs were characterized by their characteristic growth pattern and by their expression of scleraxis (SCX), tenomodulin (TNMD), thromspondin 4 (TBSH4), and surfaces markers (CD90+/CD29+;CD45‐/CD31‐). hTDCs were cultured in Dulbecco's Modified Eagle's Medium (DMEM/F‐12 with Glutamax, Gibco‐BRL) supplemented with penicillin (100 U mL^−1^), streptomycin (10µg mL^−1^) (both Sigma‐Aldrich) and 10% fetal calf serum (Gibco‐BRL) in an incubator set at 37 °C with 5% CO2 environment and subcultured to passage 2–3 before use. Culture media was changed every 2–3 days.

### Electrospinning

P(VDF‐TrFE) 75/25 wt% (Solvay Solexis) was dissolved in a 3:2 volume ratio of dimethylformamide (DMF)/acetone (Sigma‐Aldrich) at a polymer‐solvent concentration of 15–35% w/w. Briefly, a 10 mL plastic syringe (Luer lock) with a 27‐gauge stainless steel needle was placed 6 cm away from the collector, the flow rate was constant at 1 mL h^−1^, voltage 25 kV, and the collector speed was set at 3700 rpm. The collector charged with −6 kV and lower humidity levels (40–50%) yielded more favorable results. PTFE followed a two‐step process. First, 240 milligrams of poly(ethylene oxide) (PEO, *M*_w_ = 300.000,Sigma‐Aldrich), was mixed with 10 mL of PTFE, (60 wt% dispersion in H2O, Sigma‐Aldrich). After polymer blending for 2 h, the air from the syringe was removed using a centrifugation tube at 1500 rpm for 2 min and filtered (Merck Millipore) into a clean syringe at least twice to ensure no precipitates were present. The syringe was placed 10–12 cm away from the collector, and the flow rate was pre‐set at 1.25 mL h^−1^, collector speed was 2000 rpm and a voltage 12 kV was applied between collector and tip. Finally, PTFE mats were collected and annealed at 385 °C for 5 min with a heating rate of 2 °C min^−1^.

### Fibronectin Functionalization of Electrospun Scaffolds

Functionalization method was adapted from ref. ^[^ ^30^ ^]^. Briefly, oxygen plasma cleaner (900 W) was applied for 30 s to PVDF/TrFE and 30 min to PTFE. After, surfaces were immersed in a 20% v/v AAc aqueous solution at 90° to initiate the polymerization of acrylic acid (AAc).Then, the treated surfaces were rinsed with distilled H20 overnight and carboxyl groups were activated with 0.1 m EDC and 0.1 m NHS (1:1) for 1 h. Immediately after surfaces were immersed in solution containing either: Collagen I(Sigma‐Aldrich, 50 µg mL^−1^), poly‐l‐lysine (PLL) (Sigma‐Aldrich,10 µg mL^−1^) or fibronectin (FN) (Sigma‐Aldrich, F1141‐1MG, 20 µg mL^−1^).

### X‐Ray Diffraction (XRD)

X‐ray diffraction patterns from films were collected by using a Philips Xpert PRO automatic diffractometer operating at 40 kV and 40 mA, in a theta–theta configuration, secondary monochromator with Cu‐K‐α radiation (λ = 1.5418 Å) and a PIXcel solid‐state detector (active length in 2θ, 3.347°). Data were collected from 5° to 70° 2θ (step size 0.026° and time per step = 347 s) at RT. A fixed divergence and anti‐scattering slit giving a constant volume of sample illumination were used. The signal deconvolution was evaluated using the peak‐fit option of the WinPLOTR program. The profile fitting procedure (XRFIT calculation) uses pseudo‐Voigt functions with a global full width at half maximum (FWHM and a global eta (proportion of Lorentzian), and a linear background. Each peak is characterized by its position, intensity, FWHM, and eta shifts concerning the global parents. The average size of the crystalline domains β (coherently diffracting domains) of the samples was extracted from the broadening of the signal using the Scherrer equation:

(1) $$\begin{array}{c} \beta_{hkl}=\frac{k\;\cdot \;\lambda }{L_{hkl}}\;\cdot \;\cos\theta \end{array}$$

Where *β_hkl_* is the broadening of the diffraction line measured at half the maximum line intensity (FWHM) taking into account instrumental contribution (β_Inst_ = 0.1°), λ is the X‐ray wavelength, *L_hkl_* is the crystal size, and θ is the diffraction angle. *k* is the Scherrer shape factor (*k* = 0.9 was used for the calculations).

### Differential Scanning Calorimetry (DSC)

The thermal transitions of the PVDF‐TrFE scaffolds were determined on a DSC Q200 (TA Instruments). Samples of 8–10 mg were heated from −10 to 200 °C at 10 °C min^–1^. This first scan was used to determine the melting temperature (*T*_m_) and the melting enthalpy (Δ*H*_m_), as well as the Curie temperature (*T*_c_) and the Curie enthalpy (Δ*H*_c_). After this first scan, the samples were quenched in the DSC and a second scan was collected from −10 to 200 °C at 10 °C min^–1^. For non‐isothermal crystallization studies, the films were heated at a constant heating rate (30 °C min^−1^) from room temperature to 260 °C and held there for 2 min to eliminate the residual crystals and memory effects due to thermal history, and then cooled to room temperature to crystallize at the same cooling rate under a nitrogen environment. For isothermal crystallization kinetics, the films were heated at a constant heating rate of 30 °C min^−1^ from room temperature to 260 °C and held there for 2 min to eliminate the thermal history, then the melt was cooled at the same rate up to 148 °C and kept constant at 148 °C for 10 min until the sample completely crystallized.

### Fourier Transform Infrared (FTIR)

Infrared spectra of PVDF‐TrFE scaffolds were recorded on a Nicolet AVATAR370 Fourier transform infrared spectrophotometer (FTIR) operating in the attenuated total reflectance (ATR‐FTIR) mode. Spectra were taken with a resolution of 2 cm^–1^ and were averaged over 64 scans. Using the absorption band of α and β phases at 532 and 846 cm^−1^, the fraction of β phases were calculated using the following equation The β‐phase content *F*(β) was calculated by the following Equation (1):

(2) $$\begin{array}{c} F(\beta )=\frac{X_{\beta }}{X_{\alpha }+X_{\beta }}=\frac{A_{\beta }}{1.26A_{\alpha }+A_{\beta }} \end{array}$$

where AEA is the absorbance value at 841 cm^–1^, *A_α_* is the absorbance at 764 cm^–1^, and the factor 1.26 is the ratio of absorption coefficients at 841 cm^–1^ (*K*_841_ = 7.7 × 104 cm^2^ mol^−1^) to 764 cm^–1^ (*K*_764_ = 6.1 × 104 cm^2^ mol^–1^) at the respective wavenumber.

### Mechanical Properties

Tensile analysis of scaffolds was performed using a Zwick/Roell Z010 with a 1 kN load cell with a crosshead speed of 500 mm min^−1^ and a maximum extension of 500%. Thin scaffolds were cut per ASTM D 882 type specimen. Results are presented as average values of *n* = 5 replicate experiments with standard deviation.

### Fiber Alignment

The samples were mounted onto SEM stubs using double sided conductive tape and then were coated with gold/ palladium (≈10–20 nm) using a SEM specimen coating system. The samples were viewed by Hitachi S‐4700 cold field emission gun scanning electron microscope (CFE‐SEM) running at 5 kV and spot current of 5 μA. Fiber alignment was analyzed by OrientationJ plugin from ImageJ (free download from NIH).

### Voltage Outputs

To analyze the electromechanical behavior of the scaffolds, a custom made rig was developed to monitor applied strain and induced voltage using an electrometer (Keithley 6514) with virtual infinite input impedance. The Keithley electrometer was connected to the scaffold with a triaxial cable possessing three inputs (high, low, and ground). However, leakage resistance and capacitance can appear between the inputs and affect the measurement precision by introducing an offset ramp. In this case, a plausible solution was to use guarding to eliminate these effects. In this mode, the electrometer drives a current through the conductive sheet surrounding the sample inducing the same potential as the high input terminal. With both ends at the same potential, current leakage is not possible. The electrometer used here can operate in guarded mode or un‐guarded mode, as appropriate. Finally, LabVIEW was used from National Instruments to collect the data and a mechanical loading reactor to apply strain to the samples following a sinusoidal signal at different frequencies and fixed strain.

### *d*_33_ Measurements PFM

The piezoelectric response of the PVDF‐TrFE samples was investigated using piezoresponse force microscopy (PFM) (Bruker Nano Inc, Santa Barbara, CA, USA). For PFM imaging, a Pt‐Ir coated conductive probe (SCM‐PIT) with a spring constant of 2.8 N m^−1^ and a resonant frequency of 75 kHz was used. The amplitude of the detected piezoelectric signal is related to the piezoelectric coefficient (*d*_33_) of the material, whereas the phase of the signal reflects the direction of the polarization of the domains. An average of ten different locations on one sample was used to compute the average value of piezo‐response amplitude. Before imaging the PVDF‐TrFE samples, the periodically poled lithium niobate (PPLN) test sample was used for standard PFM imaging verification. The vertical piezoresponse was calibrated using deflection sensitivity of the AFM cantilever tip obtained from the force‐displacement curve.

The *d*_33_ measurement procedure is described below:

(3) $$\begin{array}{c} d_{33}=A\times s/G\times V \end{array}$$

where *A* is Amplitude, *s* is deflection sensitivity (114 nm V^−1^), *G* is vertical deflection gain (16×) and applied *V* is AC bias (0.3 V).

### Mechanical Loading Bioreactor

Briefly, cells were cultured on control and experimental scaffolds at a density of 7.500 cells per cm^2^ and left for 24 h to attach. Uniaxial mechanical loading was provided via a UniMechanoCulture T6, Cellscale bioreactor. Cells were kept under static (4% strain) or dynamic conditions for 8 h per day of 4% strain (2 mm) at 0.5, 1, and 2 Hz for 1 day, 5 days, and 10 days. A total of six scaffolds were used for each sample (replicates, *r* = 6).

### Immunostaining

All samples were fixed in 4% formaldehyde for 15 min at RT. After that, permeabilization buffer was added and incubated at 4 °C for 5 min. Samples were then blocked with 1% BSA in 1×PBS at 37 °C for 5 min. Primary antibody TNMD (1:20, ab203676, Abcam) and rhodamine–phalloidin (Invitrogen, Thermo fisher, 1:500) diluted in 1% BSA in PBS were added and incubated at 37 °C for 2 h. Finally, samples were washed with PBS/0.5% Tween‐20 for 5 min. Secondary antibody (Alexa Fluor 488, Invitrogen) diluted in 1% BSA in PBS was added and incubated at 37 °C for 1 h. Samples were washed again with 1×PBS/0.5% Tween for three times (5 min each). DAPI ((4′,6‐diamidino‐2 phenylindole, Invitrogen) was added and samples were covered with coverslips. Images were processed using ImageJ (Version 1.48, USA).

### RT2‐qPCR Array Processing

RNA was extracted using a trizol and chloroform precipitation method and purified using a Qiagen column. RNA quality after purification was assessed using an Agilent 2100 Bioanalyzer and RNA 6000 Pico Kit for low sample quantities (Agilent Technologies, USA) providing data on the integrity of RNA (RNA Integrity Number; RIN) and RNA concentration. RNA concentrations above 20 ng µL^−1^ and RIN >9 were used for subsequent conversion into cDNA. Following the manufacturer's instructions, cDNA was synthesized by reverse transcription of RNA using the RT2 First Strand Kit (SA Biosciences). PCR was performed in iQ5 Thermal Cycler (Bio‐Rad, Munich, Germany), using diluted cDNA as template (10 µL of cDNA, 10 µL of Genomic DNA Elimination Mixture and 91 µL of RNase free water) and the RT2 SYBR Green qPCR Master Mix (SA Biosciences), according to the manufacturer's guidelines. Each experiment was done in triplicate using the two donors (*N* = 2), and all samples were analyzed in technical duplicate for each gene set. Quantification results were normalized against stable housekeeping genes (TOP1, GAPDH, HSP90AB1, RPLP0, and TBP), using the geometric mean of the threshold cycle (*C*_t_) of the genes included in each plate. Expression changes were calculated using the ΔΔ*C*_t_ method and followed by calculation of regulation fold changes.

### Generation of Dot Plots

The ProHits‐viz tool was used for generating dotplots to visualize gene expression across all samples.^[^ ^57^ ^]^ The color of each “dot” represents the log2(FoldChange) of gene expression for each group sample relative to the control sample. The size of each dot represents its relative expression among other genes, in other words the bigger is the size the higher is the expression level and the rest of dots are scaled proportionately. High‐confidence values were represented by a red outline corresponding to a *p*‐value <0.05 or by a black outline corresponding to a *p*‐value <0.01, whereas for *p*‐values >0.05 the outline was faint.

### Protein Expression

Protein antibody microarray was custom made. Nexterion slide H microarray slides were purchased from Schott AG (Mainz, Germany). Alexa Fluor 555 carboxylic acid succinimidyl ester was obtained from Life Technologies (Carlsbad, CA, USA). Protein samples were labeled with Alexa Fluor 555 carboxylic acid succinimidyl ester according to manufacturer's instructions. The excess label was removed, and the buffer was exchanged with PBS, pH 7.4, by centrifugation through 3 kDa molecular weight cutoff filters. Absorbance at 555 and 280 nm was measured for labeled samples and calculations were performed according to manufacturer's instructions using an arbitrary extinction coefficient of 100 000 and molecular mass of 100 000 to enable quantification of relative protein concentration and label substitution efficiency. All commercial antibodies (Table 1) were buffer exchanged into PBS and quantified by a bicinchoninic acid (BCA) assay. Antibodies were diluted to print concentration in PBS and printed in six replicates on Nexterion H amine‐reactive, hydrogel‐coated glass slides using a SciFLEXARRAYER S3 piezoelectric printer (Scienion, Berlin, Germany) under constant humidity (62% +/− 2%) at 20 °C. Each feature was printed using ≈1 nL of diluted antibody using an uncoated 90 µm glass nozzle with eight replicated subarrays per microarray slide. After printing, slides were incubated in a humidity chamber overnight at room temperature to facilitate complete conjugation. The slides were then blocked in 100 × 10^−3^ m ethanolamine in 50 × 10^−3^ m sodium borate, pH 8.0, for 1 h at room temperature. Slides were washed in PBS with 0.05% Tween 20 (PBS‐T) three times for 2 min each wash followed by one wash in PBS, dried by centrifugation (470 × *g*, 5 min), and then stored with a desiccant at 4 °C until use. Incubations were carried out in the dark. Microarray slides were incubated as previously described. Initially, one labeled sample was titrated (2.5–15 µg mL^−1^) for optimal signal to noise ratio and all samples were subsequently incubated for 1 h at 23 °C at 9 µg mL^−1^ in Tris‐buffered saline (TBS; 20 × 10^−3^ m Tris‐HCl, 100 × 10^−3^ m NaCl, 1 × 10^−3^ m CaCl2, 1 × 10^−3^ m MgCl2, pH 7.2) with 0.05% Tween 20 (TBS‐T). All microarray experiments were carried out using three replicate slides. Alexa Fluor 555 labeled cells lysate (10 µg mL^−1^) were incubated in two separate subarrays on every slide to confirm retained antibody performance and printing, respectively (Figure 1). After incubation, slides were washed three times in TBS‐T for 2 min per wash, once in TBS and then centrifuged dry as above. Dried slides were scanned immediately on an Agilent G2505 microarray scanner using the Cy3 channel (532 nm excitation, 90% photomultiplier tubes (PMT), 5 µm resolution) and intensity data were saved as a .tif file. Antibody microarrays were verified to remain active for at least 2 weeks after printing and all incubations were carried out within that time frame. Data extraction from .tif files was performed mainly as previously described. Data were normalized to the mean of three replicate microarray slides (subarray‐by‐subarray using subarray total intensity, *n* = 4, 24 data points). Unsupervised hierarchical clustering of normalized data was performed using Hierarchical Clustering Explorer v3.0 (http://www.cs.umd.edu/hcil/hce/hce3.html) using the parameters no prefiltering, complete linkage, and Euclidean distance. All data presented here were confirmed using at least four replicates for each of the test groups and control group. The results are expressed as the mean of the values ± standard error of the mean.

Different types of arrays were fabricated to investigated tendon regeneration or phenotype maintenance (tenogenesis), intracellular molecular pathways (signaling) or membrane proteins (receptors).

### In Vivo Animal Model

The animal care research ethics committee at the National University of Ireland, Galway and Health Products Regulatory Authority (AE19125/P055) approved all the animal procedures used in this study. Also, animal care and management followed the Standard Operating Procedures of the Animal Facility of the National University of Ireland, Galway. Animals were allowed to acclimatize for at least 7 days before any surgical procedures. Subsequently, animals were acclimated to the treadmill running for 1 week, and their behavior was analyzed. A total of 105 Female Lewis rats aged 6–8 (220g) weeks were used in this study. The animals were anaesthetized by isofluorane inhalation (5% induction reducing to 1–2% for maintenance during procedures). The right leg was shaved and swabbed with iodine to minimize the risk of bacterial contamination. An incision was created through the skin (≈1cm) from the myotendinous junction distally to the osteotendinous junction. The incision provided ample exposure to the Achilles tendon. The fascia surrounding the Achilles was transected longitudinally and carefully retracted to expose the Achilles tendon. Before implantation and tendon transection, two looped sutured were inserted at the top (muscle) and bottom (bone). After the total tendon length was measured a 3 mm defect in proximal/distal extension (at 3 mm from the calcaneus) was created using a positioning device and an 11 surgical blade, resulting in a 6 mm gap after tissue retraction. The construct was then sutured (4‐0 Ethicon) to both ends of the tendon to bridge the gap using a modified Kessler technique, and the skin was sutured. After a period of 2, 4, and 8 weeks the animals were euthanized and tendon tissue, as well as contralateral tendons, were harvested. Animals recovered for 2 weeks and were gradually exposed to treadmill running (see **Table** **6**). Briefly, the treadmill running once a week for 5 min to 30–45 min for 5 days a week.

**Table 6:** Exercise protocol used for the rat running group (treadmill)

Time		Duration [min]	Speed [m min^−1^]
Week 2	Day 14	5	8–9
	Day 15	10	9–10
	Day 16	15	9–10
	Day 17	20	9–10
	Day 18	30	9–10
Week 3	Day 19	45	9–11
	Day 20	30	9–11
	Day 21	45	9–12
	Day 22	30	10–14
	Day 23	45	10–14
Week 4		30	10–14
Week 5		45	10–14
Week 6		30	10–14
Week 7		45	10–14
Week 8		30	10–14
Week 9		45	10–14
Week 10–16		30	10–14

### Histology

Repaired tendon tissues and contralateral tendons of all groups (*n* = 7) were dissected from the proximal myotendinous junction to the distal osteotendinous junction and processed for histological analysis. The samples were fixed in 10% neutral buffered formalin (24 h), dehydrated in gradient alcohols, cleared, and embedded in paraffin blocks, as reported previously. Histological sections (6 µm thick) were prepared using microtome sectioning (Leica Rotary Microtome). In order to distinguish between scar tissue or new tendon formation, polarization microscopy and picrosirius red staining were used. Also, for descriptive histology 6 µm, thick sections were stained using Hematoxylin & Eosin stain, Masson‐Goldner's stain, Alcian Blue, O‐safranin stain, Red picrosirius stain, or Herovici's polychrome stain according to the manufacturer's guidelines. Areas of chondrification within the defect region at 4 or 8 weeks after surgery were measured using Safranin O staining and ImageJ (v1.52i). For each tissue, 3 different frontal‐longitudinal sections were analyzed (1 section of the middle tendon, 1 section ventral to this middle part, and 1 section dorsal to this middle part). For each section 5 consecutive images were captured spanning the entire length of the tissue, omitting the transition zones of original tendon stumps to regenerated tissue. The volume fraction (VV) of tendon cells were used to estimate cell proliferation. A 192‐point grid was overlaid on 40× images of H&E stained tissue sections. The number of tendon cells intersecting points of the grid was counted (P_P_), along with the total number of points on the tissue (P_T_). The volume fraction of tendon cells (VV) was calculated using the formula below:

(4) $$\begin{array}{c} V_{V}=P_{P}{\text{/P}}_{T} \end{array}$$

### Histology Scoring System

In order to highlight the effect of treadmill running and differences between groups on the progression of tendon repair and calcification, a score system was adopted previously described.^[^ ^58^ ^]^ Briefly, the macroscopic score system included measurements on stained samples (Hematoxylin & Eosin, Masson‐Goldner's stain, Alcian Blue, O‐safranin, Red picrosirius, and Herovici's polychrome) three animals per group (five sections of 6 μm per animal). A point‐based scoring system was used, and the following parameters were scored (by two scorers): ECM organization of the whole repaired tendon, cellularity, proteoglycan content, cell alignment, organization of the tendon callus, integration of constructs to the normal tissue, vascularization, degenerative changes (osteochondral), and features of inflammation. A total of three scorers were used for the scoring and the slides were blinded by one of the authors (MFY).

### Functional Recovery Analysis

An animal treadmill (Exer 3/6, Columbus Instruments) track integrated with a video‐based system was used to obtain spatiotemporal parameters of gait. The animal gait was analyzed through a clear plastic Lexan at the sides of the system consisting of a cage (50.8 cm × 50.8 cm × 33 cm) with gates placed at each end of the walkway. A digital camera (8 M pixels and 120 frames per seconds) was positioned 30 cm in front of the walkway to capture the sagittal view of the rat from the walkway. The data were analyzed and processed with Kinovea software (v0.8.27). For all groups, the system was calibrated using the same scale bar located in the image and modeled the leg motion using pairs of circle markers. The ankle, knee, hip, and MTP joint angles were measured using the automatic black ink marker feature recognition on the hip, knee, ankle, and 3rd metatarsal head at the four gait stages: initial contact, mid‐stance, pre‐swing, and mid‐swing. The spatial and temporal gait parameters analyzed were step length, angle, and cycle time. The walking speed was calculated by dividing the step length by the cycle time. Each angle joint curve was normalized by cycle time before further analysis. To determine the change on gait angle, the maximum difference in amplitude (Ampl.) between initial contact and end of swing was measured during an entire step and was calculated according to following formula:

(5) $$\begin{array}{c} \max.\left(\underline{{}^{\circ}}\right)-\min.\left(\underline{{}^{\circ}}\right)=\text{Ampl}.\left(\underline{{}^{\circ}}\right) \end{array}$$

(6) $$\begin{array}{c} \text{Functional}\;\text{Recovery}=\text{Ampl}.{\left(\underline{{}^{\circ}}\right)}_{\text{post}transection}\text{/Ampl}.{\left({}^{\circ}\right)}_{\text{prior}transection} \end{array}$$

### COMSOL Simulations

To understand the influence of the potential distribution around the scaffold during electromechanical stimulation and how this might impact the cells near the scaffold, we calculated the potential distribution of the scaffold through a finite element method using electrostatic, piezoelectric, and solid mechanics interaction modulus in COMSOL. The finite element method (FEM) analysis uses an integrated device structure consisting of a tubular‐shaped electrospun scaffold composed of PVDF‐TrFE fibers under 4% deformation (0.2 mm). The geometries and the electromechanical properties are set as the real measured values. Due to the complexity of the nanofibrous structure, we used a simplified PVDF‐TrFE model consisting of a low‐density film (density of 450 kg m^−3^). The young modulus is 60 MPa, Poisson's ratio is 0.42, and the strain‐charge coupling matrix is *d*_31_ = 23 pC N^−1^, *d*_32_ = 8pC N^−1^, and *d*_33_ = −36.5pC N^−1^. In addition, we have investigated the effects of high adhesion forces (up to 30 nN^[^ ^10^ ^]^) applied through focal adhesion complexes (1–5 μm long) on a single PVDF‐TrFE fiber.

### Statistical Analysis

Statistical analyses were performed using GraphPad Prism software version 8.4.3 (GraphPad Software, CA, USA). To test whether the data was normally distributed normality tests were applied (D'Agostino and Pearson). When data followed a normal distribution a one‐way ANOVA analysis for the comparison of means between different groups was performed. Homogeneity of variances was tested using Bartlett's tests, and in a case of unequal standard deviations (SD), Welch ANOVA test was applied. If data were not normally distributed, the comparison of medians between different groups was assessed by non‐parametric Kruskal‐Wallis test followed by Dunn post‐hoc test. Since most of the data was not normally distributed, most of the results were expressed as median as central tendency characteristic and interquartile range (IQR) or range as dispersion characteristic and, *p* values of <0.05 were considered statistically significant.

## Conflict of Interest

The authors declare no conflict of interest.

## Author Contributions

M.A.F.‐Y., M.J.B., and A.P. conceived the experiments. M.A.F.‐Y. performed the experiments. A.T., S.D., A.S., and A.L. performed research. M.K., A.P., T.S., and M.P. provided materials and expertise. M.A.F.‐Y. analyzed the data and prepared the figures. A.P. and M.J.B. provided critique and context for the data. M.A.F.‐Y. wrote the manuscript. All authors read and commented on the manuscript.

## Supporting information

Supporting Information

## Data Availability

The data that support the findings of this study are openly available in bioRxiv at https://doi.org/10.1101/2020.08.03.22786.
