# Supplementary material for: A Self‐Powered Piezo‐Bioelectric Device Regulates Tendon Repair‐Associated Signaling Pathways through Modulation of Mechanosensitive Ion Channels
Source: Adv Mater. 2021 Aug 23;33(40):2008788. doi: 10.1002/adma.202008788 (PMC11468587; doi:10.1002/adma.202008788)
Supplement: Supplementary file 1 — Supporting Information [file ADMA-33-2008788-s001.pdf]

© 2021 Wiley-VCH GmbH

ADVANCED MATERIALS

Supporting Information

for *Adv. Mater.,* DOI: 10.1002/adma.202008788

A Self-Powered Piezo-Bioelectric Device Regulates Tendon Repair-Associated Signaling Pathways through Modulation of Mechanosensitive Ion Channels

Marc A. Fernandez-Yague, Alexandre Trotier, Secil Demir, Sunny Akogwu Abbah, Aitor Larrañaga, Arun Thirumaran, Aimee Stapleton, Syed A. M. Tofail, Matteo Palma, Michelle Kilcoyne, Abhay Pandit, and Manus J. Biggs**

Supporting Information

A self-powered piezo-bioelectric device regulates tendon repair-associated signalling pathways through modulation of mechanosensitive ion channels.

Marc A. Fernandez-Yague, Alexandre Trotier, Sunny Akogwu Abbah, Aitor Larrañaga, Arun Thirumaran, Aimee Stapleton, Syed A. M. Tofail, Matteo Palma, Michelle Kilcoyne, Abhay Pandit , and Manus J. Biggs**

Design of scaffold

Supplementary Figure 1 shows differences in the alignment and mechanical properties of PVDF-TrFE scaffolds depending on the type of electrospinning (short-distance or long-distance electrospinnig)

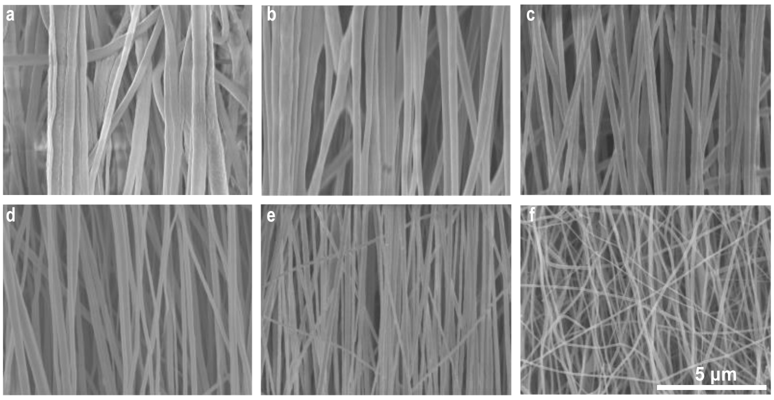

A

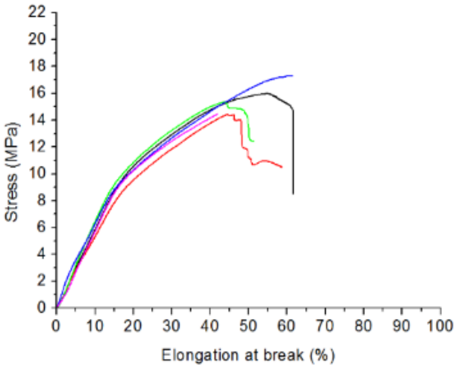

B

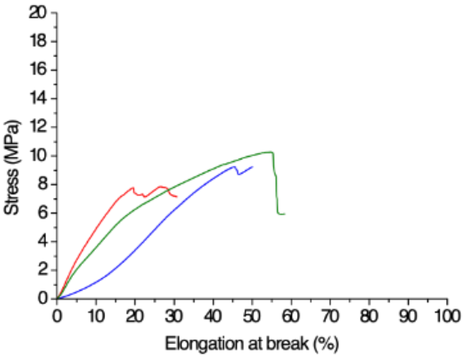

C

Supplementary Figure 1. (A), Fibres obtained with different polymeric solution concentrations (1.7, 1.4, 1.2 mg/ml respectively) using either long-distance (a,b,c) or short-distance (d,e,f) electrospinning. Fibre diameter and alignment of fibres showed dependency with polymeric solution concentration**. (B)**, Tensile mechanical test results for short-distance and **(C)**, long-distance electrospun scaffolds. Results showed that short distance scaffolds have superior elastic modulus, yield stress, elongation and reproducibility of than long-distance scaffolds.

To decouple the effect of piezoelectric stimulation from mechanical loading on the cellular response, a non-piezoelectric scaffold which maintained the topography (i.e. fibre diameter and organisation) and was chemically analogous (i.e. a fluorinated polymer) to PVDF-TrFE was also fabricated (Supplementary Figure 2) . PTFE or PE can be used as non-piezoelectric control device with similar chemical structure and composition to PVDF-TrFE. One major distinction between PE and PTFE chemical structures is the change of C-C chain flexibility (Supplementary Figure 2). PE has a planar zigzag conformation offering high flexibility to the structure. Conversely, PTFE offers some stereochemical constraint. In crystal form, the chemical conformation of PTFE always results in a helix whereas in PVDF-TrFE due to the ability of chain rotation it sometimes forms helices from the planar zigzag. To attain piezoelectric behaviour, chain rotation for dipole orientation to achieve inherent dipole moment is essential. Also, the partial charges between the Carbon and Fluorine atoms ($C^{\partial+}$—$F^{\partial-}$) attract, generating the 'strongest bond in organic chemistry' (100kcal mol^{-1} bond strength)[1]. For these reasons PTFE is exceptionally chemically stable and exhibits no piezoelectric effect. Direct electrospinning of PTFE is not possible. PTFE is a high-molecular-weight polymer, and the only practical solvent would be a low molecular weight polymer of itself such as a perfluorinated solvent (PFC) containing only C-F bonds and C-H bonds. Due to the strong C-F bond (melting point of 327ºC), dissolving PTFE and obtain a conductive polymeric solution using an organic solvent is not possible. First, a stable dispersion (surfactant content 7.2% wt

%) of 160 nm PTFE particles (62 wt%, pH 10.5) was electrospun in combination with a fibre forming agent, polyethene oxide (PEO). Aligned PTFE nanofibres were obtained using a ratio between 0.01 PTFE:PEO. Secondly, the scaffolds were annealed at a temperature beyond the melting point (395ºC) producing high-quality and continuous PTFE fibres. As observed in Supplementary Figure 2, resulting mechanical properties of PTFE scaffolds showed low ultimate strength that does not correspond to values in the literature and it can be attributed to defects presents in the fibres due to PEO decomposition. Furthermore, DSC measurements showed a clear single peak of a highly crystalline phase (ΔXc= 61.1 %, PTFE). FTIR and XRD measurements further confirmed the PEO removal. After sintering, only peaks at 1201 and 1145 cm^{-1} in the FTIR spectra corresponding to C-F bonds from PTFE were obtained. XRD measurements showed four peaks, a unique dominant peak at 18.2 and peaks at 31.8, 37.1 and 42.2 that correspond to the same fingerprint of pure PTFE powder

A

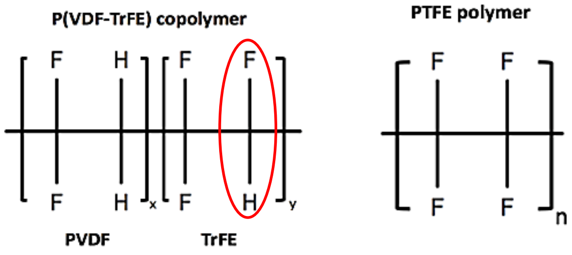

B

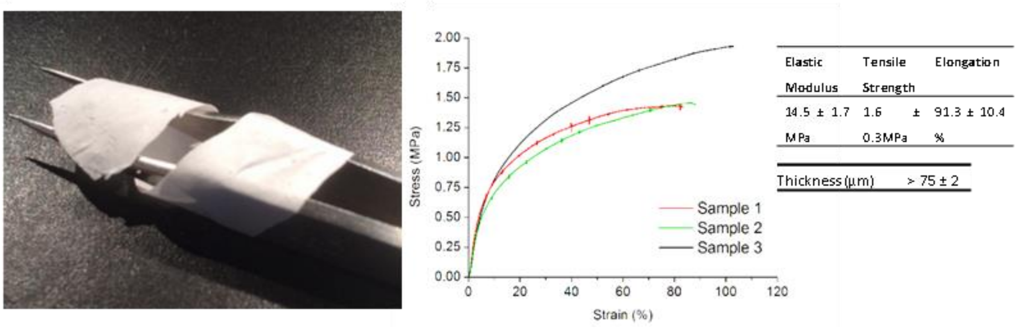

Elastic Modulus	Tensile Strength	Elongation
14.5 ± 1.7 MPa	1.6 ± 0.3MPa	91.3 ± 10.4 %

Thickness (µm)	> 75 ± 2

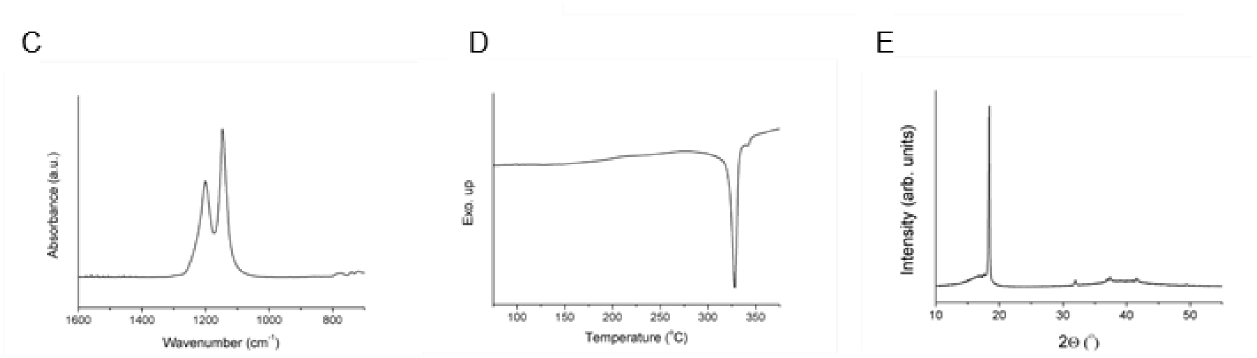

Supplementary Figure 2. (A) Schematic of the molecular structure of PTFE polymer and P(VDF-TrFE) copolymer. Note the structure of PTFE with four polar covalent Carbon—Fluorine bonds making it extremely stable and therefore Non-Piezoelectric. **Note:** Within the P(VDF-TrFE) copolymer, the -TrFE component exhibits a strong dipole across the Hydrogen to Fluorine bond (red circle). Mechanical properties of PTFE. (B), Mechanical propeties of PTFE scaffolds. **(C)**, FTIR measurement showing C-F bond. **(D)**, DSC measurement showing the melting point at 330 C **(E)** XRD measurement (**F**)

There are several advantages of using extreme rotating-collector linear speeds during electrospinning (~30 m/s). First, it enables the formation of mesoscopic joints between adjacent fibres as a result of residual (not evaporated) solvent that maximise the mechanical properties of the scaffold. Limited evaporation is a function of short flight times and use of high boiling point solvents (i.e. DMAC) as described by Persano et al. in similar studies[2]. And secondly, individual fibres resulting in finer (fibre diameter) and more crystalline fibres (Supplemenraty Table 1) increases the overal device piezoelectrical performance. The use of DMAc in combination with acetone (50:50 v/v) allowed the surface morphology of the fibres to be slightly rough and promotes cellular attachment in combination with an ECM protein functionalisation of the surface.

Supplementary Table 2 shows the results from analysing the FTIR spectra of PVDF-TrFE scaffolds. The bands that are exclusively associated with β-phase were discerned (i.e., bands at 840 cm-1 and 1279 cm-1). However, bands associated with the α-phase (i.e., bands at 764 cm-1 and 976 cm-1) did not appear in the spectra, suggesting that the β-phase is the predominant crystalline phase in the PVDF-TrFE scaffolds. The degree of crystallinity was calculated by

deconvolution of XRD spectra into crystalline peaks and amorphous halo. (Supplementary Figure 3 and Table 3). The samples showed different degree of crystallinity (40-49%) and similar average size of the crystalline domain (~10 nm). Scaffolds spun using long-distance (between collector and needle) showed the lowest β-phase content (58%) compared to short-distance spun scaffolds+annealing (78%).

The thermal transition properties of P(VDF-TrFE) scaffolds were evaluated using differential scanning calorimetry (DSC) (Supplementary Table 3). PVDF-TrFE scaffolds demonstrated two main transitions: an initial endothermic peak, associated with the Curie temperature (Tc ~117ºC) and a second endothermic peak that is associated with the melting temperature (Tm~141ºC). The observed ΔHc and the ΔHm values were ~5 J g-1 and ~17 J g-1 for the scaffolds without annealing. This process modified the thermal properties, and a new endothermic transition occurred at 90ºC (related to the polymerisation of acrylic acid, AA, during ECM functionalisation steps), and the ΔH_m value decreased to ΔH_m=12.74 J g^{-1} (Supplementary Table 3). This change is associated with the presence of fibronectin bound to the scaffold

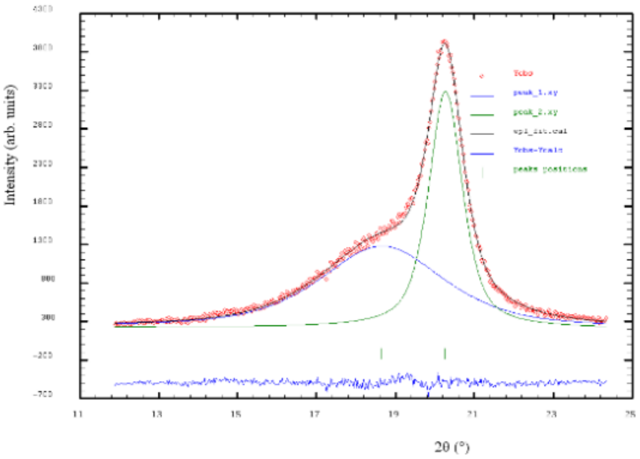

Supplementary Figure 3. Deconvolution of the XRD spectra into crystalline phase and amorphous halo of PVDF-TrFE scaffolds.

Supplementary Table 1. Degree of crystallinity and the average size of the crystalline domain of the PVDF-TrFE scaffolds.

Sample ID	*Deg.crystallinity %*	*Crystalline size domain*
Long-distance (random).	*40*	9
Long-distance (aligned).	*43*	9
Short-distance (aligned)	*49*	9
Short-distance (aligned) + annealing	*47*	11

Supplementary Table 2. β-phase content of the PVDF-TrFE scaffolds in function of processing determined by FTIR.

Sample ID	Beta phase content / Total
Long-distance	58
Short-distance	63
Short-distance + annealing	78

Supplementary Table 3. DSC measurements results. Effect of distance and annealing during surface functionalisation on the thermal properties of PVDF-TrFE scaffolds.

Sample ID	*Tc*	*ΔHc*	*Tm*	*ΔHm*
Long-distance (aligned).	117	5.2	140	17.4
Short-distance (aligned).	117	5.4	140	17.1
Short-distance + annealing (aligned).	117	3.4	139	12.7

In general, thermal annealing (over Tc temperature 132ºC for 2h) resulted in highly crystalline samples (>70%). The increase in crystallinity after annealing originates from increased γ-phase (15% on annealed samples compared to 1% on as-spun samples). However, under SEM visualisation (data not shown), it was noticed that thermal annealing resulted in significant loss of fibre organisation and introduced defects on the fibre surfaces (in the form of cracks) due to thermal expansion coefficient changes between crystalline and amorphous phases. It was obtained that annealing at 90ºC for 1-hour process showed a significant increase in the crystallinity without affecting fibre structure and surface. Higher annealing temperatures (i.e.110 to 135) had a negative impact on essential characteristics of the samples (fibre organisation and surface morphology, increased brittleness) for the indented tissue engineering application (decreased porosity as fibre form several mesoscale and macroscale joints between the fibres and the total volume of scaffold was reduced by 30%).

Finally, the scaffolds were stretched to obtain an increase in stiffness and finer fibre diameter. The diameter of fibres was measured by SEM and showed a reduction from 540 to 516 nm after cold drawing at 12 % strain and correlates well with the theoretical values :

$$V_o = \pi . l_o . d_{o2} / A \qquad \text{Eq. 1}$$

Considering the volume constant and an elongation of 12% the reduction coefficient was 0.95, and the calculated fibre diameter was 513 nm, close to the experimental value.

<u>*In vitro* characterisation</u>

As shown in Supplementary Figure 4, Oxygen (O1s) and Nitrogen (N1s) XPS characteristic peaks confirmed successful fibronectin coating on the scaffold surface. The spectrum of the C1s Carbon signal (see Figure 11) at 284.6 V revealed changes in the C-C and C-H bond energy suggesting new covalent bonds have been formed. The other three contributions are related to intra-chain polymer bonds C-F. Finally, the evident Fluor signal present on both spectra suggests that the modification with fibronectin is in the nanoscale range (< 20 nm).

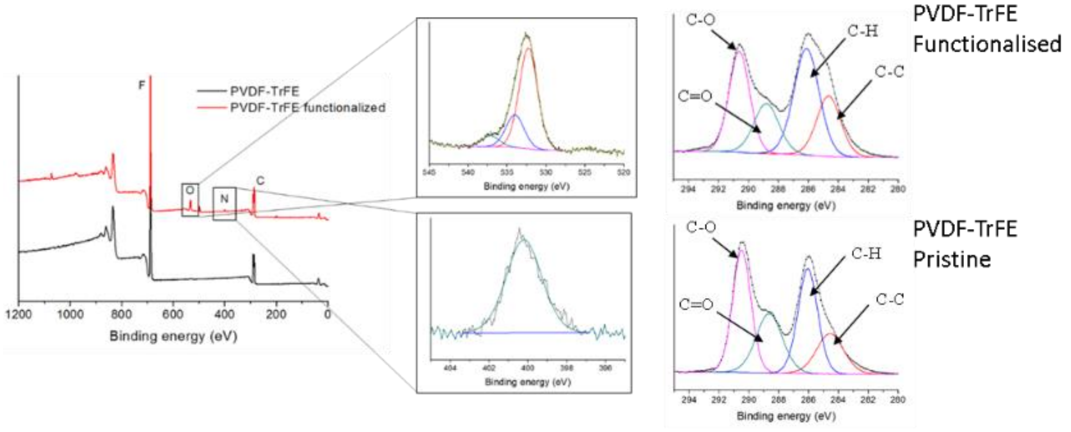

Supplementary Figure 4. XPS measurements after fibronectin surface functionalisation. Surface chemistry analyses. X-ray Photoelectron Spectroscopy (XPS) spectra are shown for surfaces following fibronectin surface immobilisation (in red) and compared to pristine surfaces without surface functionalisation (in black). High-resolution spectra are showing the presence

of peaks corresponding to Nitrogen (N1s at 405.5 eV) and Oxygen (O1s at 530.9 eV). Surface chemistry analyses. X-ray Photoelectron Spectroscopy (XPS) high-resolution spectrum for Carbon (C1s) showing typical hydrocarbon contamination with C==, C-C, C-H and C-O components. Carbon signal was deconvoluted for a) pristine and b) functionalised scaffolds showing significant changes in C-C component.

Interestingly, we observed that cell growth and cell viability was susceptible to other mechanical loading parameters such as duration, displacement and frequency (. We found that cells became more senescent at higher frequencies (>1 Hz) relative to low frequencies (<1Hz). Under continuous mechanical stimulation of scaffolds, it was observed that cell proliferation was reduced and proportional to the loading frequency. Similarly, others have shown that longer stimulation duration and higher frequency of dynamic stretching results in decreased cell proliferation of tendon cell cultures[11].

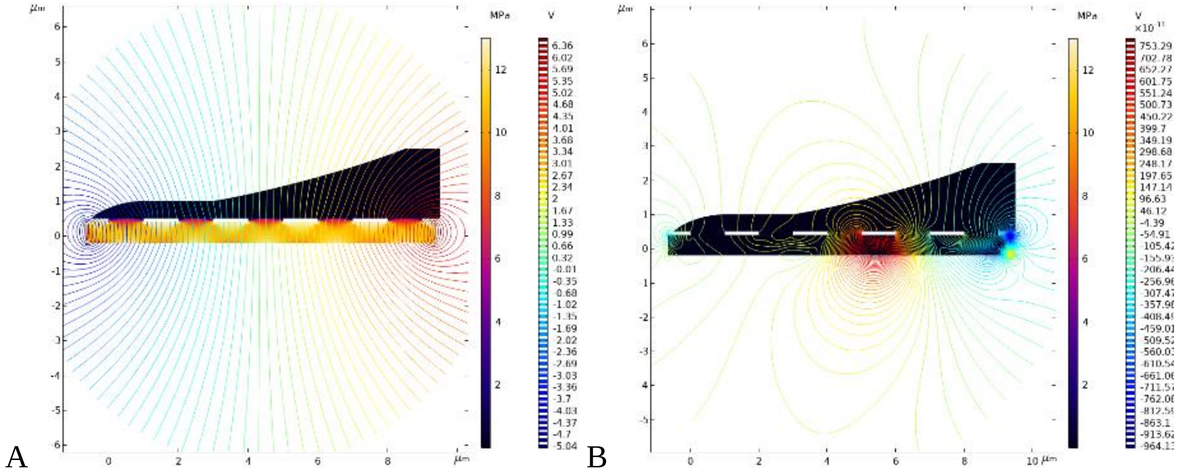

Supplementary Figure 5. COMSOL Simulations showing generated von Misses Stress and electrical potential distribution on a single fibre due to (A) 4% strain or (B) focal adhesion mediated cell tension. The stress-activated piezoelectric signals during EMS (4% strain) are higher orders than the electric signals generated by cell adhesion.

A final stimulation frequency of 0.5 Hz was chosen based on an analysis of cell survival under dynamic conditions (Figure 4). It was obtained that hTDCs that lost their elongated morphology (Supplementary Figure 6) underwent significant down-regulation in TNMD and SCX

expression when cultured on 2D planar films. Conversely, when dedifferentiated hTDCs were cultured on 3D electrospun scaffolds, they maintained a spindle-like morphology and demonstrated increased TNMD and SCX expression relative to planar 2D films. Stretching of aligned scaffolds resulted in increase elongation of cells as observed by higher nuclei aspect ratio (Supplementary Figure 6) relative to static controls.

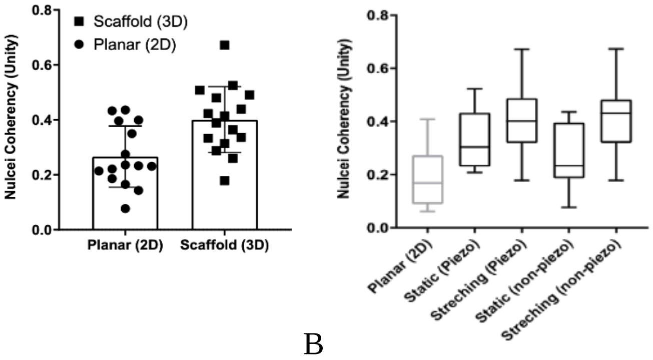

Supplementary Figure 6. (**A**) Comparison between planar and fibrous (aligned fibres) morphologies on nuclei coherency (aspect ratio/deformation) and (**B**) Comparison between static and dynamic (stretched 4%) cultures of hTDCs using piezoelectric and non-piezoelectric scaffolds on nuclei coherency (aspect ratio/deformation).

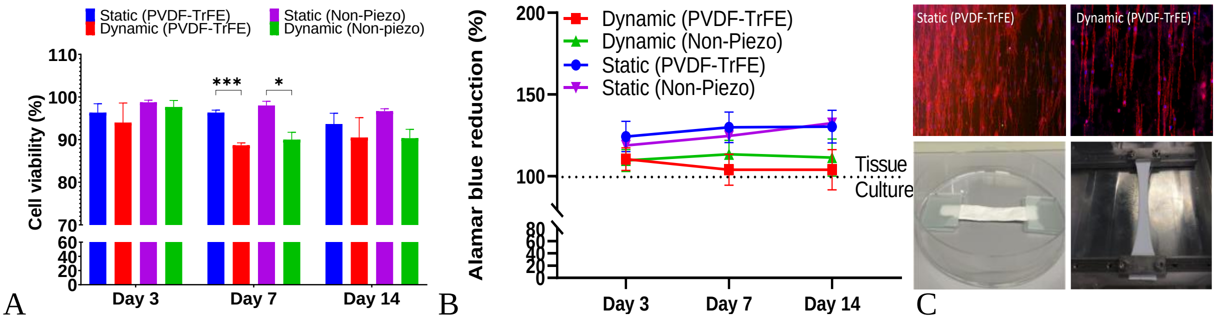

Supplementary Figure 7. (A) Cell viability and **(B)** Cell proliferation (Alamar blue) for cells exposed to static and dynamic conditions cultured on either piezoelectric or non-piezoelectric scaffolds. **(C)** hTDCs stained for rhodamine phalloidin (actin fibres) cultured on scaffolds in static and dynamic conditions. Data expressed as mean ± SEM. *p<0.05, **p<0.01, ***p<0.001, two-way ANOVA. N=3 donors, replicates=3 scaffolds per group.

A custom-made gene array was designed to assess the gene expression of focal adhesion, collagens, receptors (integrins, ion channels and growth factor receptors) and, inflammation

and assess cell biological functions such as differentiation towards bone, cartilage or tendon. Gene analysis was performed on cells cultured on non-piezoelectric and piezoelectric scaffolds under mechanical stimulation (0.5 Hz, 8 hours/day) at days one, five and ten. SCX and MKX were upregulated at days ten and ten and are involved in collagen synthesis and fibrillogenesis. Collagen I, III and IV were also upregulated at days five and ten under electromechanical stimulation (Supplementary Figure 8).

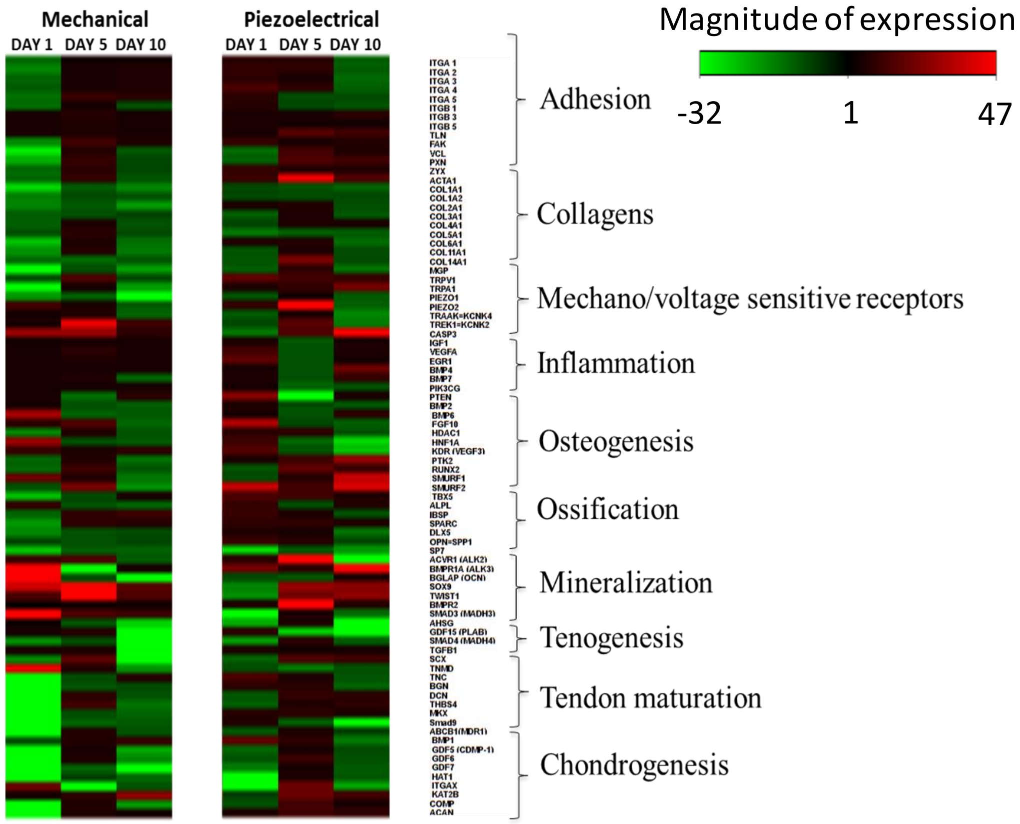

A

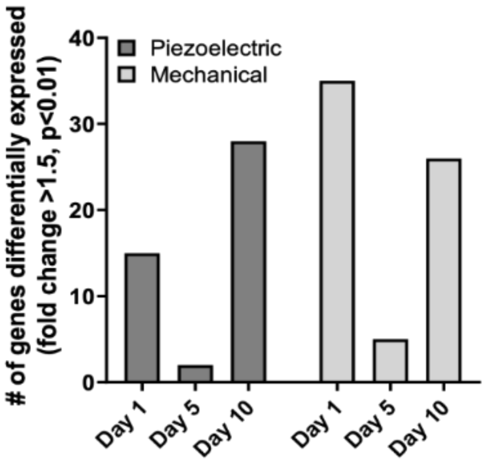

B

Supplementary Figure 8. **(A)**, Gene expression arrays for mechanical and piezoelectric stimulation. **(B)**, Comparison of genes differentially expressed for mechanical and piezoelectric stimulation at days one, five and ten respectively.

hTDCs subjected to mechanical stimulation exhibited down-regulation of the integrin signalling pathway (Figure 5), with exceptions observed in cytoskeleton rearrangement. Down-regulation of vinculin (VCL) was prominent with mechanical stimulation as was the gene associated with the linker protein paxillin (PXN). Conversely, up-regulation of VCL and PXN was observed in hTDCs subjected to electromechanical stimulation showed cytoskeleton rearrangement and high expression of tendon specific genes. Protein expression using a custom-made antibody microarray was used as a validation method to confirm the differential modulation of osteospecific pathways under EMS and MS (Supplementary Figure 9). The expression of tendon-related proteins TNMD, TNC and BGN, were upregulated under EMS but downregulated under MS at day ten. The activation osteospecific signalling pathways β-catenin and BMP were investigated through the phosphorylation of SMAD1/5/8 (BMP signalling) and active β-catenin. As shown in Figure 5, EMS resulted in downregulation of BMP signalling but upregulation of functional proteins including TNMD, SCX, TNC and BGN.

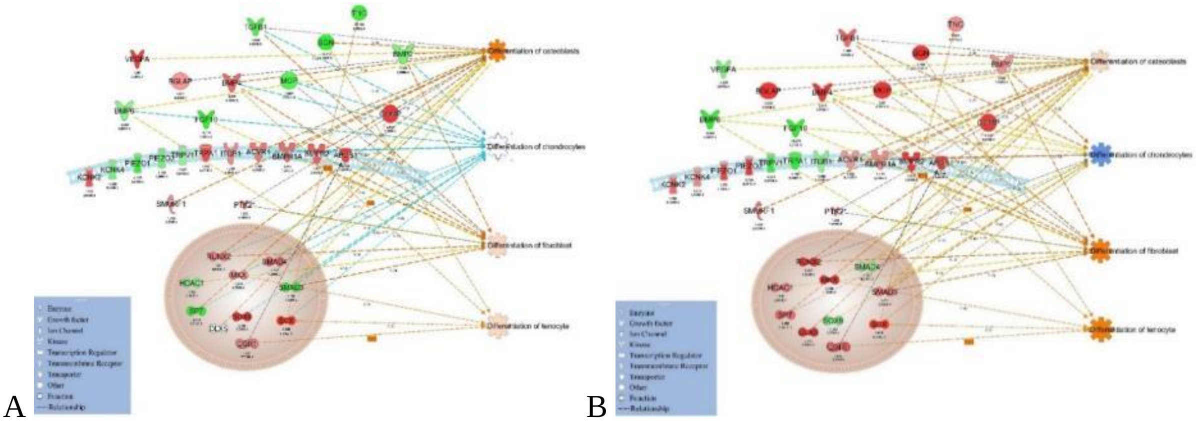

A B

Supplementary Figure 9. Ingenuity pathway analysis. **(A)**, Mechanical and **(B)**, electromechanical (piezo) stimulation for ten days induced modulated cell function, including differentiation. Ingenuity pathway analysis identified a mechanistic network of 37 genes which underwent statistically significant modulation. Network analysis of the identified genes:

recently discovered mechanoreceptors Piezo1 and Piezo 2 indicated a positive correlation with changes in gene expression and increased tenospecific function.

In vivo characterization

A modified kessler-technique was used to reattach the ends of the injured tendon using sutures and generating a gap of 6 mm. The gap was either left uncover or covered by a the device (Supplementary Figure 10).

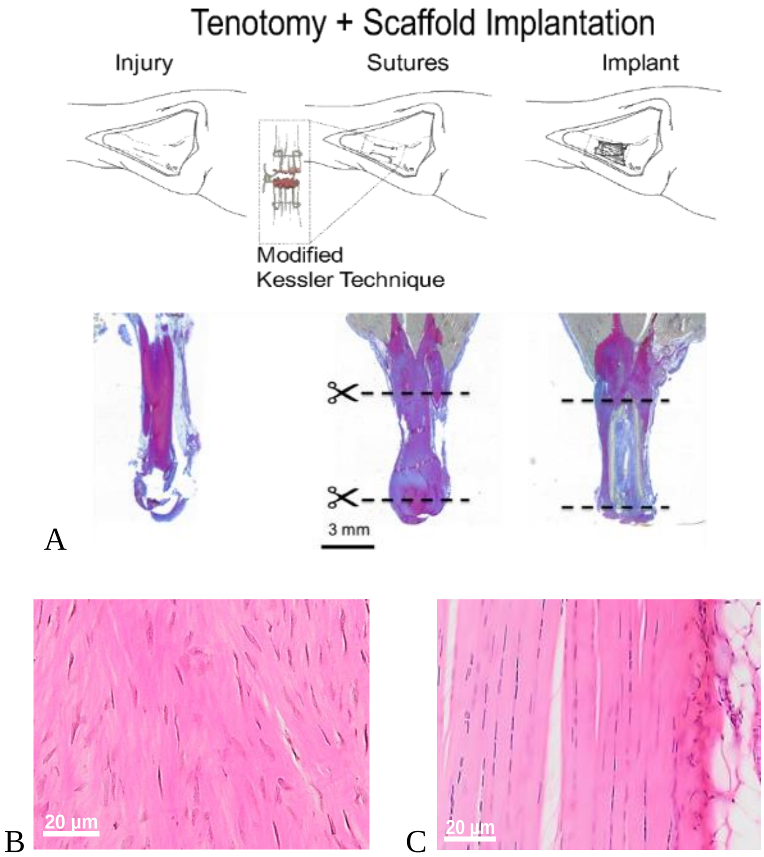

Supplementary Figure 10. (A), Schematic representation and representative Herovici's stain histological images of both the intact tendon and injured tendon, repaired with either suture or with an electrospun scaffold. **(B)**, H&E images of injured tendon sample (1 week) and **c**, intact tendon sample showing differences in cell morphology. Pink, tissue matrix; purple, cytoplasm; blue, nuclei (Scale bar 20 and 10 µm respectively).

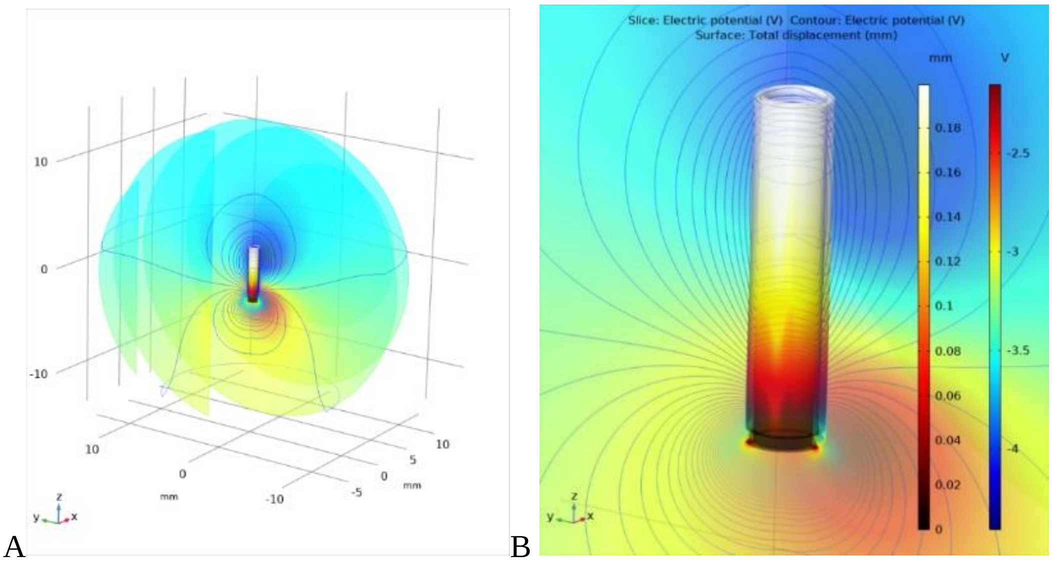

Supplementary Figure 11. (A) Simplified COMSOL model of a tubular-shaped scaffold under 4% deformation and electric potential distribution. **(B)** Higher magnification of the scaffold showing the displacement and electric potential fields generated by the scaffold.

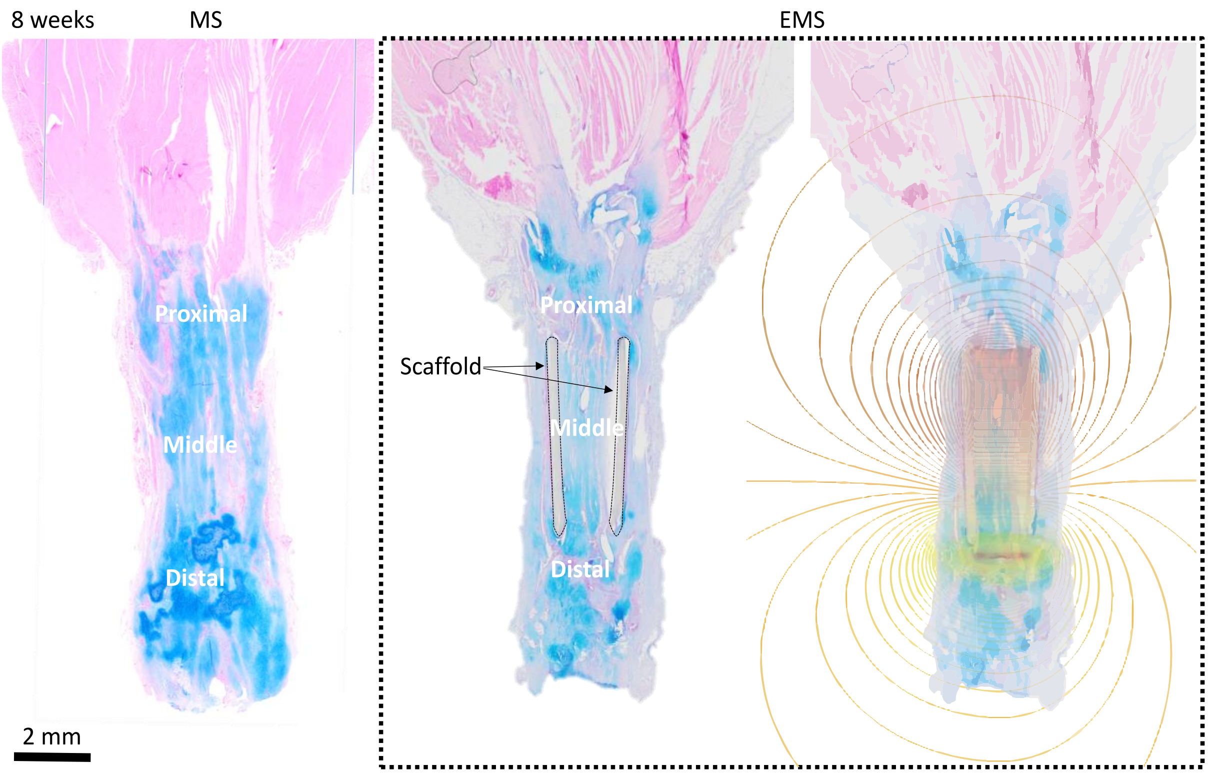

Supplementary Figure 12. Alcian blue-stained tissue samples for MS and EMS. Black arrows indicate the implanted device (scaffold) location. The electric field distribution generated by the device under 4% deformation is represented by the contour plot of the electric-field lines superposed to the Alcian blue stained sample. The connection between bone and scaffold

(distal) is more rigid than the connection between muscle and tendon (proximal) and Stress, and electric field concentrates in the distal area.

Our group [3,4] and others[5–8] have demonstrated that electrospun PVDF-TrFE is biocompatible by looking at cell viability, proliferation, and in vivo response. Our study revealed a minimum inflammatory reaction with a very thin encapsulation formation (see supplementary figure 13) around both piezoelectric and non-piezoelectric devices equally and up to 8 weeks post-implantation.

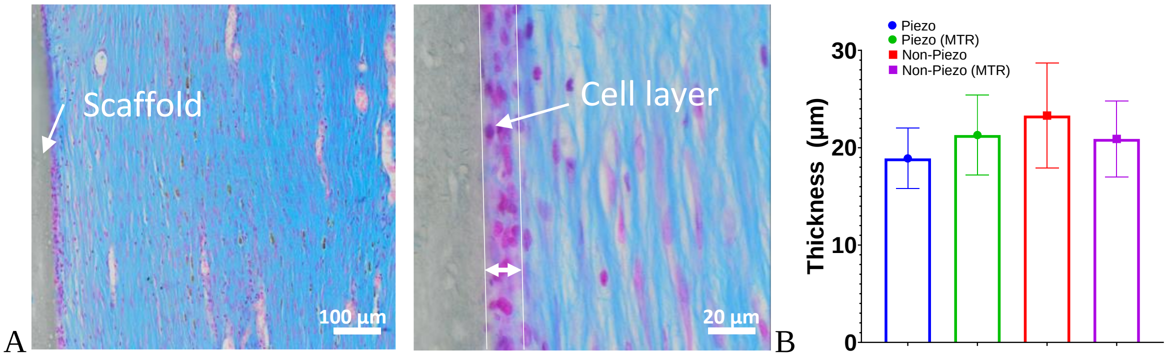

Supplementary Figure 13. **(A)** Representative images of low and high magnification of Alcian blue stained tendon sample treated with piezoelectric scaffold for 8 weeks under dynamic conditions. **(B)** Quantification of cell layer thickness for all the conditions at 8 weeks post-injury.

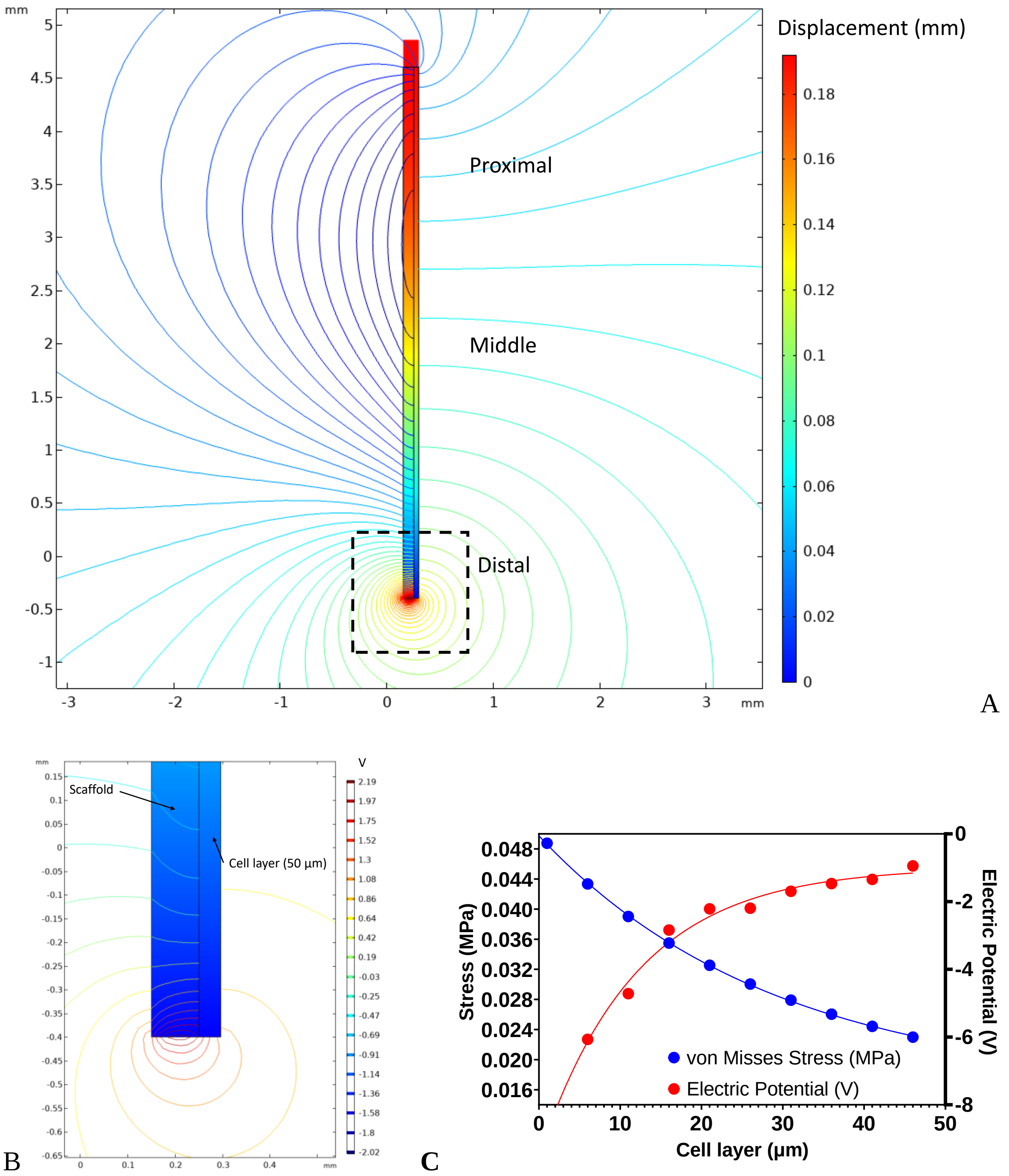

Figure 14. (A) Section view of a piezoelectric scaffold with one side demonstrating an adherent cell layer and indicating the electric potential at proximal, middle and distal regions **(B)** Potential distribution at the distal scaffold region in response to an adherent cell layer **(C)** Measurement of stress and electric potential in function of cell layer thickness.

The Functional recovery analysis results of injured tissues treated with non-piezo and piezo scaffolds (static and TR) at weeks 4 and 8 are shown in Supplementary Table 4. It was observed

that animals treated with a Piezo (MTR) and Non-Piezo (MTR) showed significant functional recovery in the ankle, MTP and knee joints after 4 and 8 weeks. Results are expressed by mean ± standard deviation, * p<005, ** p<0.01.

Supplementary Table 4. Functional recovery analysis over a period of 8 weeks in animals undergoing treadmill running. Significant differences were observed between the animals undergoing treadmill running relative to animals subjected to static conditions (N=7).

		Injury				Non-piezo				Piezo			
	Ctrl (º)	Static (4 w)	MTR (4 w)	Static (8 w)	MTR (8 w)	Static (4 w)	MTR (4 w)	Static (8 w)	MTR (8 w)	Static (4 w)	MTR (4 w)	Static (8 w)	MTR (8 w)
MTP	**100±2**	**52±5**	**48±3**	**51±7**	**71±8**	**56±5**	**58±5**	**55±9**	**92±8**	**55±5**	**48±5**	**52±8**	**92±8**
Knee	**80±3**	**53±4**	**58±7**	**63±7**	**67±7**	**55±4**	**62±3**	**55±2**	**72±3**	**56±10**	**60±7**	**53±6**	**74±6**
Hip	**18±2**	**12±4**	**15±5**	**9±4**	**15±5**	**10±1**	**14±2**	**12±5**	**15±3**	**10±3**	**8±2**	**8±4**	**12±8**
Ankle	**70±2**	**57±11**	**55 ±3**	**58±7**	**61±3**	**53±7**	**54±4**	**51±5**	**67±3**	**55 ±9**	**57±3**	**57±5**	**67±3**

After the injury, the tendon stumps were observed to retract, generating a gap that became filled with granulation tissue. The fibrous regenerating tissue was characterised by loose collagen fibre organisation and an overall heterogeneous texture after two, four and eight weeks, with increased cellularity and ingrowth of vessels, nerves, fat deposition and calcification (Supplementary Figure 15).

At 2 wpi the newly formed tissue was observed to be composed of disorganised granulation tissue comprised of collagen III. The digital analysis shows that the distribution of the orientation of fibres was broad and disorganised (Supplementary Figure 15) A circular colour map shows the correlation between orientation and colour code. The polarised image demonstrates that the deposited collagen is highly disorganised and shows poor birefringence, characteristic of collagen type III.

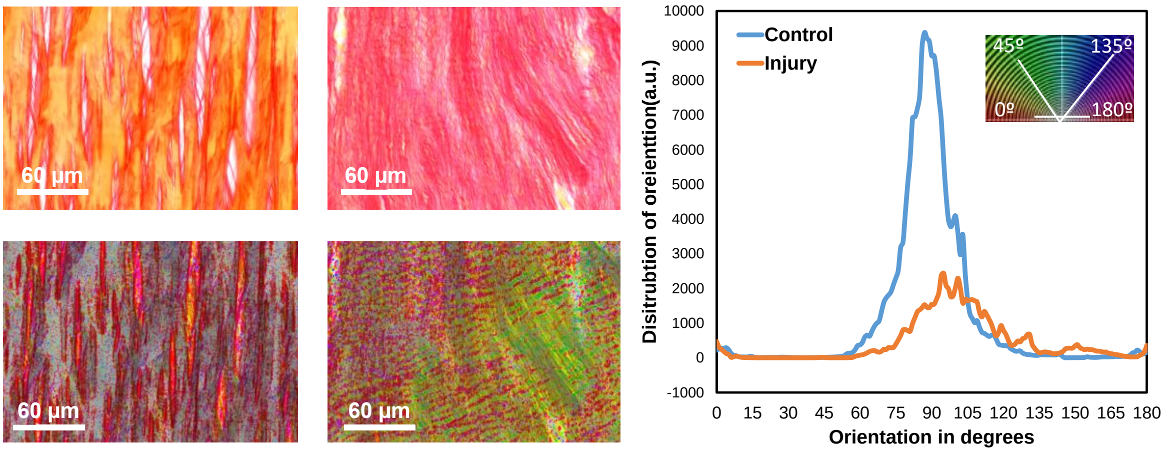

A B C

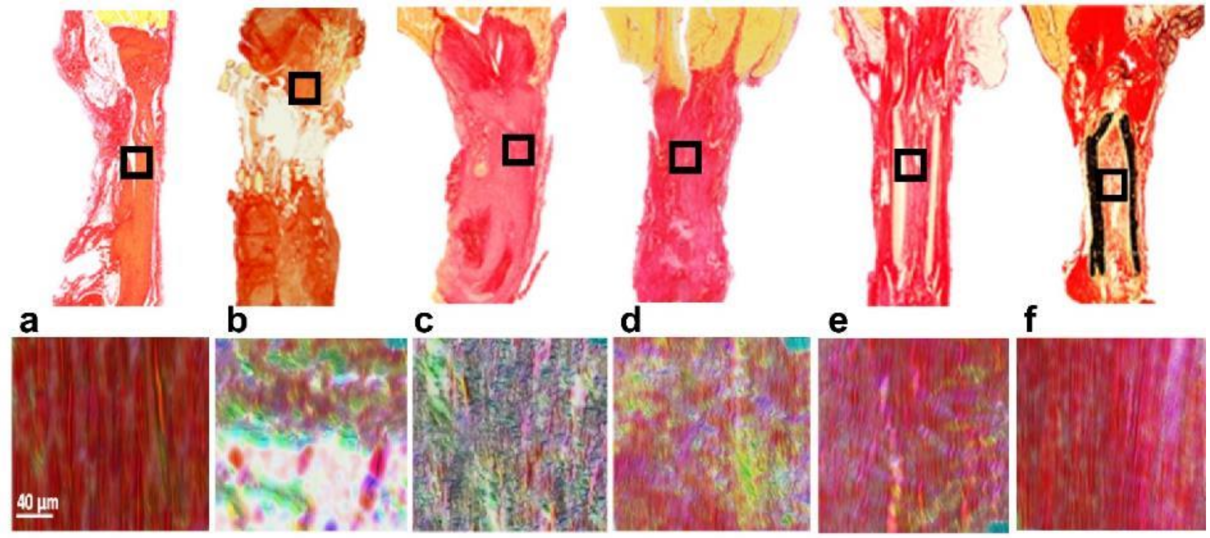

D

Supplementary Figure 15. (A), Picrosirius red-stained tendon histological sections 1-week post tendon transection without (left) and with polarisation (right) for contralateral intact tendon tissue and **(B),** injured tendon tissues. **(C),** The digital analysis shows that the distribution of the orientation of fibres was broad and disorganised. A circular colour map shows the correlation between orientation and colour code. The polarised image demonstrates that the deposited collagen is highly disorganised and shows poor birefringence, characteristic of collagen type III. **(D),** Picrosirius red staining of intact, suture-repaired and scaffold-repaired tendon tissue. Orientation distribution analysis using high-magnification images (box region) indicated that tissue organisation was increased in animals treated with piezo and non-piezo scaffolds. a) intact tendon, b) sutured tendon 24 hours post-injury, c) 1-week post-surgery and d) 8 weeks post-injury; e) tendon repaired with piezoelectric scaffolds 8 weeks post-injury and f) tendon repaired with a non-piezoelectric scaffold 8 weeks post-injury

At 2 wpi, the tendon tissue composition was highly heterogeneous, possessing an increased cellular content and noted ingrowth of nerves and blood vessels relative to earlier time-points (data not shown). At 2 wpi, irregular voids were present between the large collagen type I fibres which were filled with granulation tissue and cells demonstrating a chondrogenic phenotype. At 4 wpi, a condensation of these cells showing a chondrogenic phenotype in both injury and non-piezo scaffolds treated animals was observed. This process, initiated by mechanical compression and the hypoxic conditions experienced by resident and infiltrating cells, was pronounced at the bone and tendon insertion areas. Indeed, by eight weeks post-injury endochondrial ossification was prominent in all static (cage) groups (Supplementary Figure 16) .

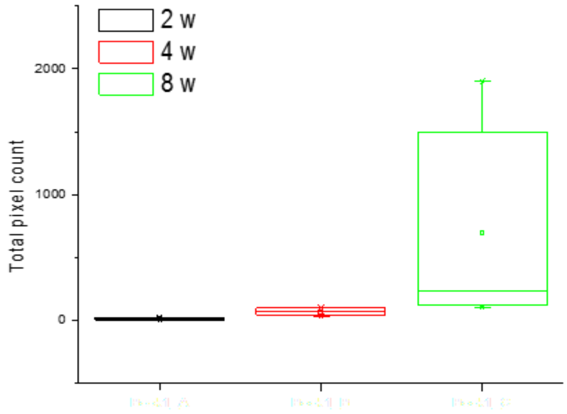

Supplementary Figure 16. Quantification of the chondrification area for all samples after 2, 4, and 8 wpi. Histomorphometric analysis indicates a significant increase in chondrification eight weeks post-injury.

Following the inflammation phase (2 wpi), tendon structure reorganisation was initiated, and areas of high cell density were observed close to the tendon stumps endings. Cartilage formation first appeared at the distal stump region. By 4 wpi, many cartilage nodules were formed in the region of the granulation tissue close to the distal stump ending. Within this region, the chondrocytes matured into hypertrophic chondrocytes and mineralisation of the tissue was initiated. Several small cartilage nodules were present from four weeks both at the tendon

stumps and throughout the granulation tissue. Progressively, cartilage nodules served as template for new bone formation via endochondral ossification, and ectopic bone formation resulted from hypertrophy of chondrocytes and blood vessel infiltration (Supplementary Figure 17).

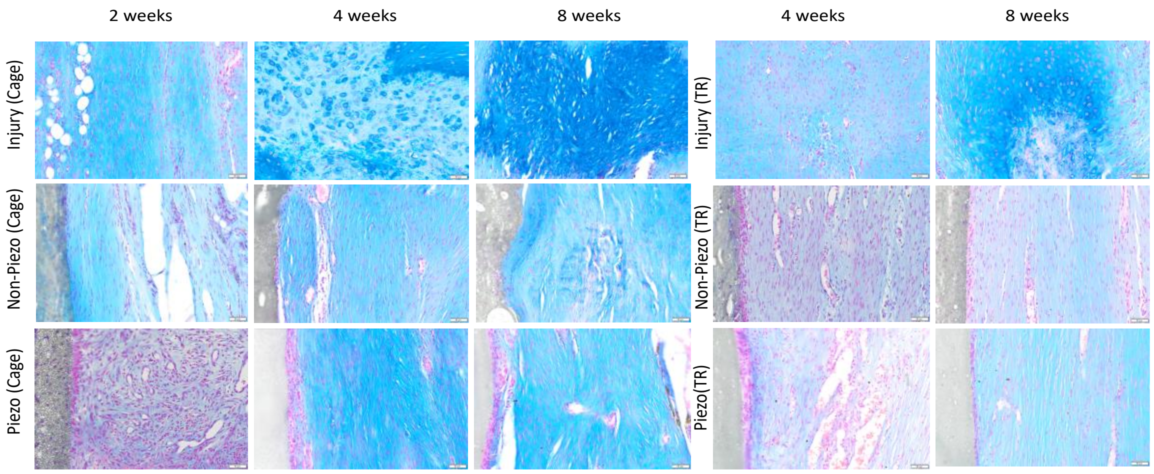

Supplementary Figure 17. Alcian-blue stained representative histological images indicating the role of electromechanical stimulation on proteoglycan deposition in animals subjected to static or treadmill running conditions. Tendon repaired with piezoelectric and non-piezoelectric scaffolds showed less intense proteoglycan (GAG) content. Conversely, transection injuries treated with a suture possessed highly disorganised tissues with increased cell proliferation, increased vasculature and fat deposition, and/or an increased GAG content. Scale bar is 200 µm.

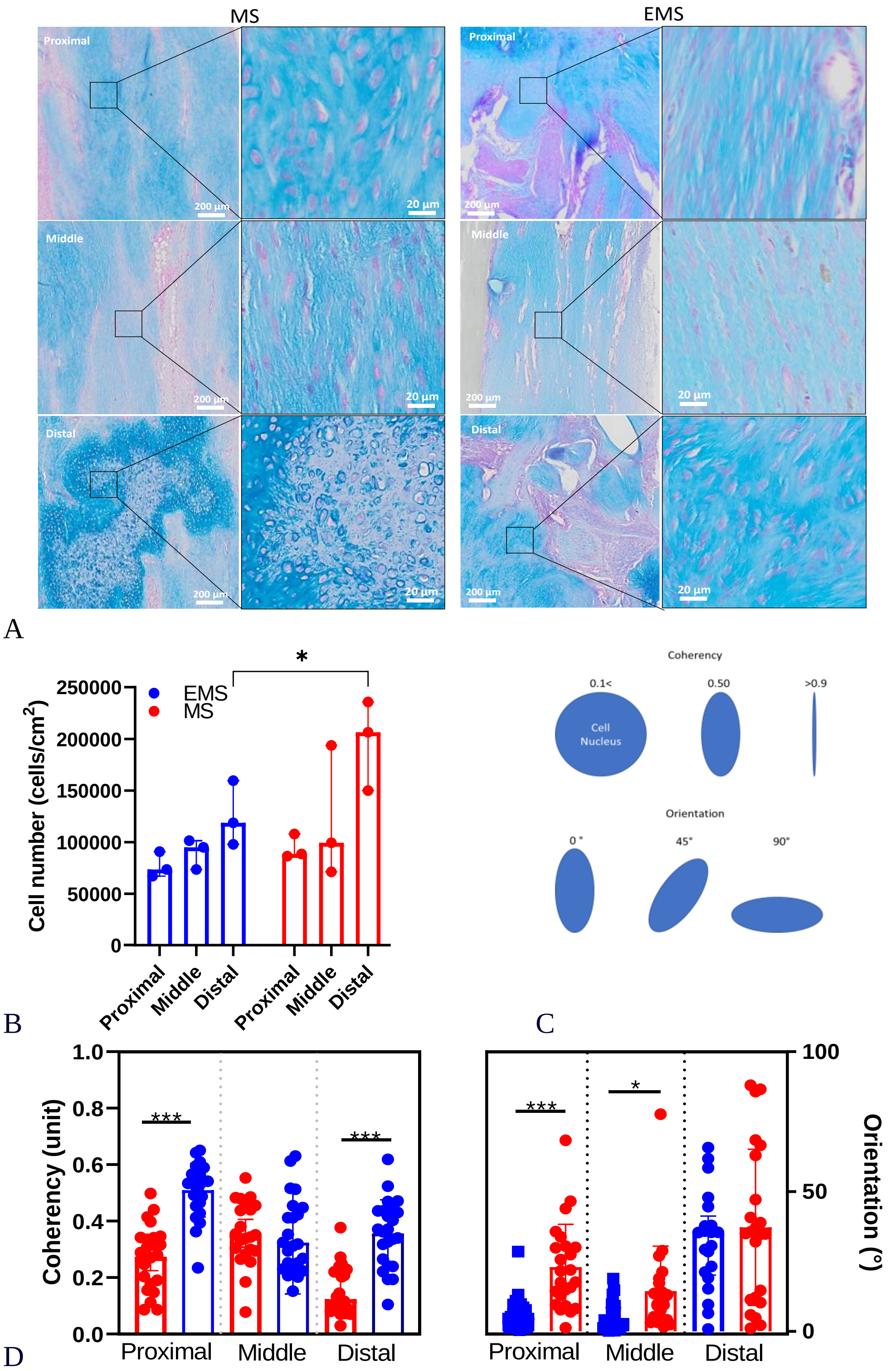

Supplementary Figure 18. (A) Higher magnifications of Alcian blue stained samples for MS and EMS at proximal, middle and distal areas showing the differences in cell number and morphological changes. (B) Quantification of cell number (cells/cm^2) at the different locations (proximal, middle and distal) (C) Schematics describing the morphological parameters. Coherency is defined by inverse of the aspect ratio of the cell nucleus. The more elongated the

cell is, higher the coherency value. Orientation is calculated by the angle of deviation with a fibre at 90 degrees. When cells are aligned with the fibre direction, the orientation value is 0. (D) Quantification of cell morphological parameters, Coherency and Orientation, at proximal, middle and distal locations. Data expressed as median ± IQR. *p<0.05, **p<0.01, ***p<0.001, Kruskal-Wallis test. N=102 cells, replicates=4 animals per group.

In order to evaluate the degree of tendon repair and the effect of treadmill running on tendon ECM morphology, organisation and biochemistry, histomorphometric measurements on stained histological sections from three animals (5 slides in total per animal) were employed at eight weeks post-injury. A point-based composite scoring system was employed to grade tissue repair using parameters based on 1) the cell number relative to intact tendon 2) the tissue calcification relative to intact tendon 3) the tissue vascularisation and innervation relative to intact tendon 4) the extent of fat deposition relative to intact tendon 4) the fibre orientation relative to intact tendon 5) the cellular morphology relative to that of cells from to intact tendon. The results of the composite scoring system are shown in Supplementary Table 5.

Supplementary Table 5. A composite scoring system was employed for the histological evaluation of repair at eight weeks post-injury. Values are expressed in Mean ± SEM of scored points. 0-25 % (4 points); 26-50 % (3 points); 51-75% (2 points) and 76-100% (1 point).

	Parameters						
Group	Cell number	Calcified area	Vessels and nerves	Fat deposits	Fibre orientation	Cell shape (aspect ratio)	Total
Control	4 ± 0	4 ± 0	3.5 ± 0.5	3.5 ± 0.5	3.5 ± 0.5	4.0 ± 0	**23**
Injury	1 ± 0.5	2.0 ± 0.5	3.5 ± 0.5	2.5 ± 0.5	1.5 ± 0.5	2.0 ± 0.5	**12.5**
Injury (TR)	1 ± 0.5	2.5 ± 0.5	2.5 ± 0.5	2.5 ± 0.5	2.5 ± 0.5	2.5 ± 0.5	**13.5**
NonPiezo	1 ± 0.5	2.0 ± 0.5	3.0 ± 0.5	2.5 ± 0.5	2.5 ± 0.5	1.5 ± 0.5	**12.5**
NonPiezo (TR)	2 ± 0.5	2.5 ± 0.5	2.5 ± 0.5	2.5 ± 0.5	2.5 ± 0.5	2.0 ± 0.5	**14.0**
Piezo	1 ± 0.5	1 ± 0.5	2.5 ± 0.5	2.5 ± 0.5	2.5 ± 0.5	2.5 ± 0.5	**12.5**
Piezo (TR)	1.5 ± 1	3.0 ± 0.5	2.5 ± 0.5	2.5 ± 0.5	3.5 ± 1.25	2.5 ± 0.5	**15.5**

Collagens are the main bulk constituent of tendon tissue. During wound healing, early granulation tissue is produced by fibroblasts to act as a temporary scaffold for cell attachment and proliferation. Generally, granulation tissue is mainly comprised of collagen III, and low levels of collagen I. As the healing continues, the ratio of collagen I / collagen III becomes higher but generally never reaches its original values (~1.4). As shown in Figure 6C, a significant amount of collagen type III was observed at 4 wpi for all MTR groups and for Piezo(MTR) group returned to baseline 8 wpi (~1.3). An increase in the synthesis of collagen I over collagen III is a good indicator of tendon tissue maturation. Conversely, Collagen II represents almost 80% of the total collagen content in cartilage.

In our study, we found that increased expression of the Piezo2 and TRPA1 ion channels at 2 and 4 wpi (Figure 6E) were obtained in tendon tissues exposed to MS. The increased expression of these ion channels was positively correlated with the activation of the signalling pathways ERK/MAPK and FAK ($p<0.01$) at week four (Supplementary Figure 19).

MAPK/ERK FAK

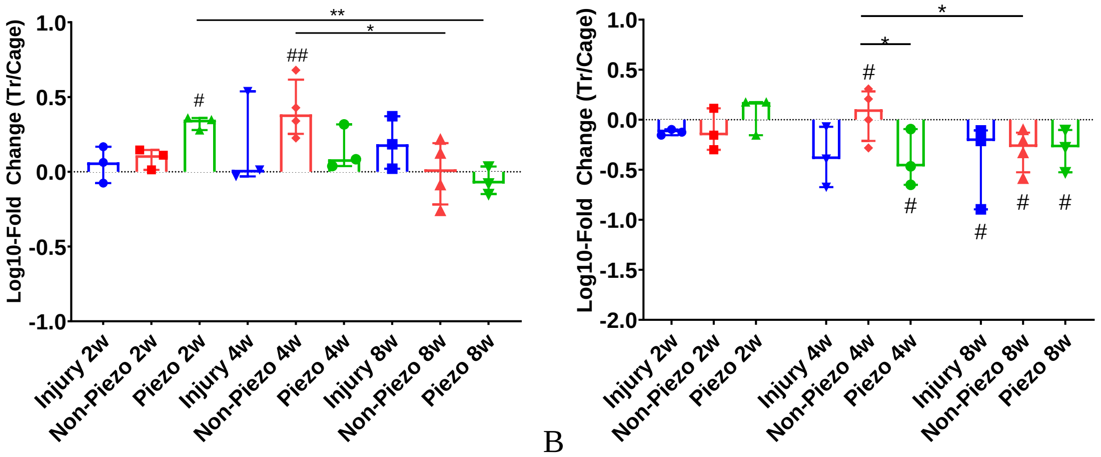

A B

BMP WNT

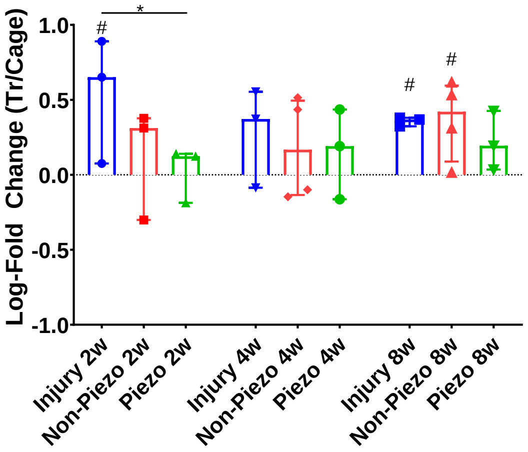

C

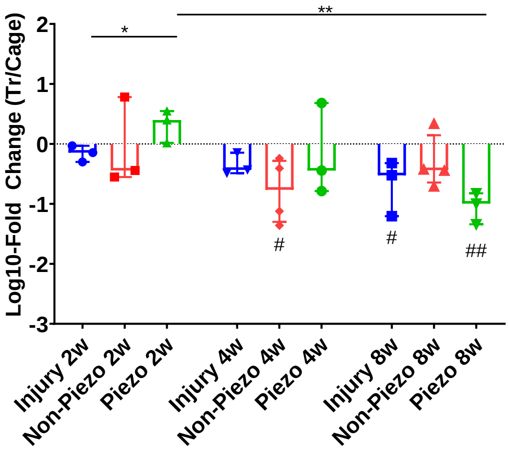

D

Supplementary Figure 19. **(A)**, Protein Microarray data showing Log10-fold change in MAPK and **(B),** FAK expression in tissues derived from TR animals relative to tissues derived from cage animals for each experimental group at weeks four and eight. Significance is shown between samples and week two (cage only). **(C)**, Protein Microarray data showing log-10 fold changes in BMP and **(D),** Wnt expression in tissues derived from MTR animals relative to tissues derived from cage animals for each group at weeks four and eight. Significance is shown between samples and week two (cage only). Analysis performed using Kuskal-Wallis (N=4) with post-hoc Dunn (*,# p<0.05. **, ## p<0.01).

It is largely recognised MAPK/ERK signalling pathway is activated during exercise and represents the link between exercise and adaptive changes in tendon composition. In addition, MAPK/ERK signalling pathway plays a central role in mediating cell division, migration and survival. The activation of the MAPK/ERK pathway was always observed in all (MTR) groups relative to cage groups. The upregulation of FAK signalling was significant only for MS at 4 wpi and downregulated at 8 wpi (p<0.01) (Supplementary Figure 19). The molecular pathways associated with the ectopic formation of bone after injury were studied, and significant upregulation in BMP signalling was observed in caged and MTR groups treated with sutures (Injury) and non-piezoelectric scaffolds (MS) at 8 wpi (p<0.05). The activation of the Smad1/5/8-dependent pathway (BMP) was significantly increased in Injury and MS groups at 8 wpi compared to their corresponding static group (p<0.05). The activation of BMP signalling pathways is involved in the homeostasis of the native tendon and during normal (healthy tendon) structural adaptation; however, excessive activation can lead to ectopic bone formation.

The main associated upregulated proteins with Injury (MTR) and MS groups were integrins β1, BMPR1A, Collagen II, SCX, and Tenomodulin.

References

[1] D. O'Hagan, *Chem. Soc. Rev.* **2008**, DOI 10.1039/b711844a.

[2] L. Persano, C. Dagdeviren, Y. Su, Y. Zhang, S. Girardo, D. Pisignano, Y. Huang, J. a Rogers, *Nat. Commun.* **2013**, *4*, 1633.

[3] M. A. Fernandez-yague, C. Vallejo-giraldo, *Electr. Act. Mater. Med. Devices* **2016**, *12*, 167.

[4] A. Poudel, M. A. Fernandez, S. A. M. Tofail, M. J. P. Biggs, *Front. Chem.* **2019**, DOI 10.3389/fchem.2019.00364.

[5] Y.-S. Lee, G. Collins, T. Livingston Arinzeh, *Acta Biomater.* **2011**, *7*, 3877.

[6] G. G. Genchi, L. Ceseracciu, A. Marino, M. Labardi, S. Marras, F. Pignatelli, L. Bruschini, V. Mattoli, G. Ciofani, *Adv. Healthc. Mater.* **2016**, *5*, 1808.

[7] S. M. Damaraju, Y. Shen, E. Elele, B. Khusid, A. Eshghinejad, J. Li, M. Jaffe, T. L. Arinzeh, *Biomaterials* **2017**, *149*, 51.

[8] Y.-S. Lee, S. Wu, T. Livingston Arinzeh, M. B. Bunge, *Biotechnol. Bioeng* **2017**, *114*, 444.
